# Recent advances in hydrogels for treating periodontal diseases and oral mucosal diseases

**DOI:** 10.3389/fbioe.2025.1605672

**Published:** 2025-08-04

**Authors:** Yu Gui, Yunpeng Zhang, Haoyang Xu, Wanli Yang, Haitong Huang, Mengqin Gu, Yu Sun

**Affiliations:** ^1^ School of Stomatology, Hainan Medical University and Hainan Academy of Medical Sciences, Haikou, Hainan, China; ^2^ Chongqing Key Laboratory of Oral Diseases, Chongqing Municipal Key Laboratory of Oral Biomedical Engineering of Higher Education, Chongqing Municipal Health Commission Key Laboratory of Oral Biomedical Engineering, The Affiliated Stomatological Hospital of Chongqing Medical University, Chongqing, China

**Keywords:** hydrogels, adhesive hydrogels, periodontal disease, oral mucosal diseases, therapeutic interventions

## Abstract

Periodontal and oral mucosal diseases are prevalent oral health challenges. Thanks to the natural constraints imposed by the moist and complex intraoral environment, current therapeutic modalities are significantly limited by two key issues: inefficient, low sustained drug release and short intraoral drug retention times, both of which compromise therapeutic efficacy. To address these challenges, hydrogels—a novel class of biomaterials with unique biological and physicochemical properties—have emerged as promising solutions. Hydrogels, with their 3-dimensional (3D) polymer network, are promising biological reagents, particularly for drug delivery. They achieve controlled and sustained release at target sites, and coupled to their therapeutic outcomes, their use in oral healthcare is critical. Consequently, hydrogels have been extensively investigated in both foundational and clinical research, particularly within a wound dressing context. This article systematically examines the limitations associated with conventional therapies and elucidates the mechanisms underpinning the therapeutic efficacy of hydrogels in managing different periodontal and oral mucosal diseases. Furthermore, we explore the clinical challenges and opportunities associated with applying hydrogel-based strategies in oral therapeutics, and we propose future directions for hydrogel research and development.

## 1 Introduction

As part of a healthy lifestyle, maintaining good oral health is important. However, due to inadequate personal dietary and oral hygiene practices, an increasing number of individuals now face oral health challenges (Li H. et al., 2024; Li et al., 2023a; Peres et al., 2019). Periodontal tissue diseases and oral mucosal diseases are common oral diseases. The former leads to the progressive destruction of tooth-supporting tissues, ultimately causing tooth loss, which impairs masticatory function, compromises esthetics, and reduces quality of life. Additionally, these diseases also interact with systemic diseases, including diabetes, which may aggravate periodontitis. The latter diseases can damage the oral environment, and if they worsen, may even lead to cancer (Peres et al., 2019; Xiao et al., 2017; Hajishengallis 2022; Genco and Sanz 2020; Belibasakis et al., 2024). Despite significant progress in oral disease medicine, treating oral periodontal and oral mucosal diseases still faces immense challenges due to the unique properties of oral structures and biomaterials. For instance, the humid environment in the mouth influences drug stability and adhesion, leading to significantly prolonged treatment times for periodontal and oral mucosal diseases, and also recurrent attacks that cannot be fully cured. Therefore, identifying high-performance biomaterials that address periodontal and oral mucosal diseases have considerable, practical significance.

**FIGURE 1:**
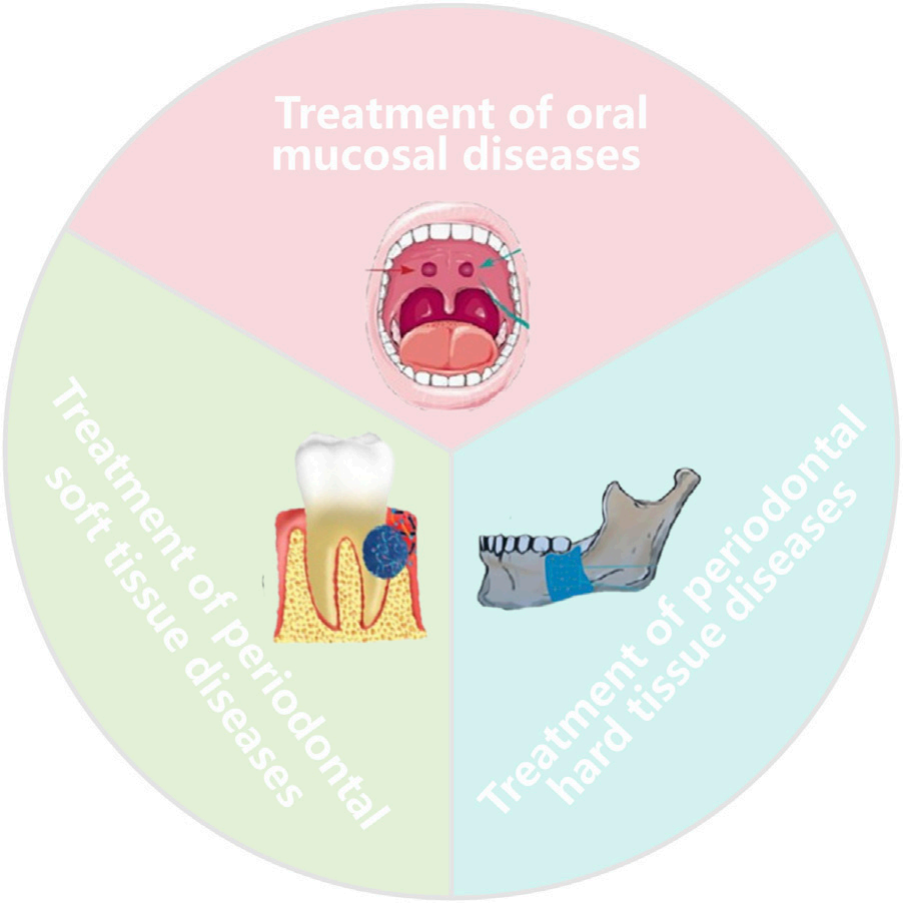
Functional hydrogel for periodontal tissue diseases and oral mucosal diseases (Li H. et al., 2024). With permission from ACS NANO. Sorce from figdraw.

Hydrogels have three-dimensional (3D) polymer chain network structures that have an immense potential in biological applications (Wang et al., 2023; Cao et al., 2021; Merino et al., 2015; Yadollahi et al., 2016). Hydrogels are significantly advantageous in terms of drug delivery, and are ideal drug carriers for targeted site control, sustained release, and evaluating therapeutic effects. Additionally, hydrogels provide enhanced bioavailability, protection, and mucosal adhesion for encapsulated or adsorbed drugs, and also have significant biological activities, including antibacterial, blood coagulation, and blood regeneration properties (Cao et al., 2021; Yang et al., 2022; Fakhri et al., 2020). In recent years, hydrogels have been extensively used to treat periodontal tissue and oral mucosal diseases, such as periodontitis, the oral mucosa, and also alveolar bone repair (Figure 1).

Current reviews have mainly focused on hydrogel applications in periodontitis or drug delivery, but ignored the limitations of current clinical trials (Dini et al., 2025; Pan et al., 2025; Zheng et al., 2023). Thus, the objective of this review is to provide a comprehensive and up-to-date summary of recent hydrogel developments in managing periodontal tissue (both soft and hard) and oral mucosal disorders. First, we characterize hydrogel properties and functions, and then briefly discuss several common periodontal and oral mucosal tissue diseases along with their clinical treatments. We then systematically explore and summarize the operational mechanisms and therapeutic outcomes underpinning hydrogels in treating various periodontal and oral mucosal tissue diseases. Lastly, we address hydrogel challenges and limitations in oral clinical practice, highlight emerging opportunities, and review their development in oral health.

## 2 The clinical status of periodontal tissue and oral mucosal diseases

At initial stages, periodontal tissue and oral mucosal diseases are characterized by soft tissue damage in local oral wounds, mostly caused by an imbalanced oral microbial community.

Periodontal tissue disease is characterized by the progressive degradation of both soft and hard tissue components in the periodontal complex (Roberts and Darveau, 2015). This destruction is mediated by an imbalanced microbial community and abnormal immune responses in the gums and periodontal tissues. Periodontal tissue diseases encompass both periodontal soft tissue and hard tissue diseases. Gingivitis, an early manifestation of periodontal soft tissue disease, is a common symptom in the majority of the global population. Periodontitis, a more severe form of periodontal soft tissue disease, represents gingivitis progression (Zini et al., 2021). The clinical treatment of periodontal soft tissue mainly encompasses basic periodontal and surgical treatments. However, mechanical periodontal soft tissue disease treatments are effective only in the short term, and often require high technical proficiency. Furthermore, these methods only generate temporary results and are not long-term solutions (Dos Santos et al., 2024; Curtis et al., 2020; Sanz et al., 2020; Usui et al., 2021). Alveolar bone resorption represents a form of periodontal hard tissue disease; it is a further consequence of ineffective periodontal soft tissue disease treatment and potentially leads to alveolar bone damage. However, the effective management of alveolar bone resorption issues remains an unresolved clinical challenge.

Oral mucosal diseases, also known as soft tissue oral diseases, encompass several conditions affecting the oral mucosa and soft tissues. Oral submucosal fibrosis (OSF), recurrent aphthous ulcers, and oral lichen planus (OLP) are the three most prevalent oral mucosal diseases. Currently, OSF treatment approaches mainly encompass drug therapy, mouth opening exercises, and elective surgery. Nonetheless, a lack of a standardized treatment protocols in clinical settings is evident, with existing therapies having restricted efficacy and a high incidence of side effects. Similarly, no definitive treatments exist for recurrent aphthous ulcers. The primary objectives of such treatments are to alleviate pain via local or systemic medication, expedite ulcer healing, and diminish the frequency and severity of ulcer episodes (Guo et al., 2023; Passi et al., 2017). OLP is an unexplained disease and typically necessitates symptomatic treatments, which involve various local and systemic medications to mitigate its signs and symptoms.

Due to the humid and highly dynamic milieu of the oral cavity, periodontal and mucosal tissue diseases, as well as oral wounds caused by the surgical treatment of both, local medication effects are usually not ideal. However, systemic medications can induce significant side effects. In light of this, identifying efficient and reliable treatments for oral periodontal tissue and oral mucosal diseases remain challenging. Thus, an urgent need exists to discover effective biomaterials and treatment technologies that address these difficulties and enhance success rates in treating these diseases (Zhang et al., 2023; Andabak-Rogulj et al., 2023; Didona et al., 2022).

## 3 Hydrogel properties and applications

Hydrogels have cross-linked polymer chains with a 3D network structure, and are renowned for their special characteristics: high water content, flexibility, porous architecture, and excellent biocompatibility. As a versatile and powerful biomaterial, hydrogels have immense potential in drug delivery, tissue engineering, biosensors, regenerative medicine, and other industries (Vedadghavami et al., 2017). Hydrogels are categorized into natural and synthetic types. Typically, natural hydrogels encompass cellulose, chitosan, collagen, alginate, agarose, hyaluronic acid, and gelatin, all of which exhibit favorable biocompatibility, bioactivity, and biodegradability profiles. However, these materials tend to have relatively weak mechanical strength and poor stability. In rare instances, certain natural hydrogel materials may act as allergens, posing potential immune risks to sensitive individuals. Synthetic hydrogels, on the other hand, are constructed from synthetic polymers, with commonly used materials, including cellulose derivatives and polyester. They have excellent mechanical strength, but some synthetic hydrogels are biologically inert, relatively hydrophobic, and have limited bioactivity. To address the deficiencies of natural hydrogels and the limitations of synthetic hydrogels, and also minimize their respective drawbacks while emphasizing their strengths, researchers have combined both to create ideal hydrogels (Ho et al., 2022; Ran et al., 2024).

Given the complexity and diversity associated with oral health issues, combined with oral milieu limitations and variability, dental biomaterials must efficiently interact with both oral soft and hard tissue types. Therefore, effective treatment methods are required for oral diseases. Among these, hydrogel biomaterials have been extensively used in dental research, from treating oral diseases to reconstructing tissues, including periodontal regeneration, jaw bone regeneration, and soft tissue healing (Chen et al., 2023). Despite considerable advantages in treating oral diseases, hydrogels have some drawbacks; their rapid degradation and poor verification regarding long-term biocompatibility challenge their transition into clinical applications.

Next, we focus on hydrogel applications in treating several typical periodontal tissue and oral mucosal diseases. We also address issues regarding future clinical applications, such as hydrogel degradation rates and biocompatibility. A detailed analysis and summary is provided (Table 1).

**TABLE 1:** Comparative analysis of hydrogel-based therapies versus conventional treatments in periodontal and oral mucosal diseases.

Comparison metrics	Periodontal diseases		Oral mucosal diseases	
	Conventional treatment	Hydrogel in periodontal diseases	Conventional treatment	Hydrogel in oral mucosal diseases
Methods for the treatment	Professional mechanical plaque removal; periodontal surgery; antibiotic treatment; bone grafting	LRG-P@*LGG* GOE1 hydrogel; hydrogel-loaded nano-hydroxyapatite	Glucocorticoids; steroids; self-limited	Sodium hyaluronate/bioglass composite hydrogel; mucoadhesive poloxamer-based hydrogels; ANSBs
Methods of delivery	Oral administration; injection; surgical implantation	Injection; percutaneous absorption	Injection; sticky cream	Injection; spray
Drug residence time	Short-term	Sustained and long-term	Short-term	Sustained and long-term
Mucoadhesive properties	Poor	High adhesion	Poor	High adhesion
Drug release mechanism	Passive diffusion	Sustained release	Diffusion	Diffusion and swelling
Side effects	Risk of resistance; additional tissue damage; immunization risks	No side effects	Depression; mental disorder	No side effects
Therapeutic effects	Limited; suboptimal induction	Complementary adjuvant traditional therapy; supportive capacity, superior inductivity	Transient efficacy and frequent relapse	Effectively mitigate fibrotic progression; reduce recovery time
Antimicrobial capacity	Additional antibiotics required (medium but short duration)	High (sterilisation rate over 90%); dual sterilisation mechanism; natural antimicrobial drug-carrying		
Treatment compliance	Poor	Improve compliance	Poor	Improve compliance; easy
Therapeutic complexity	Complex and technically demanding	Easy		
References	Sheikh et al. (2017) Sanz et al. (2020)	Pan Y. et al. (2020) Ge et al. (2024)	Louisy et al. (2024) Jones et al. (2024)	Diaz-Salmeron et al. (2021) Guo et al. (2023)

## 4 Hydrogels for periodontal tissue and oral mucosal diseases

### 4.1 Managing periodontal soft tissue disorders

Periodontitis is a common periodontal tissue disease, the periodontal tissue to the tooth body to support the fixed and bear the role of occlusal force, determines its relatively closed structure. So bacterial infections spread, the closed environment instead of isolating the inflammation area becomes a difficult problem. At the same time, the body’s immune responses react to this destruction and damage tooth body support structures (Li Q. et al., 2024), with the appearance of tooth loosening. On the one hand, protecting periodontitis areas is vital to completely isolate wounds from the oral flora and avoid wound inflammation, while on the other hand, host immune responses should be reduced to preserve periodontal tissue function. And periodontitis typing exists multiplicity, in addition to the traditional periodontitis classification into chronic and aggressive conditions. Periodontitis also exists a special group of typing, i.e., the manifestation of systemic disease. In recent years, diabetic periodontitis is better studied and the most representative. It compared with the ordinary chronic periodontitis, which has a difference in the expression level of advanced glycation end products (AGEs) and RAGE (AGE receptors), comparing to normal chronic periodontitis (Thomas et al., 2024). This may affect the course and severity of periodontal disease, as well as some differences in treatment. In a systematic study of hydrogels used for periodontal therapy, *in vivo* hydrogel efficacy was enhanced by using biologically active agents in three major areas: antimicrobial, anti-inflammatory, and regenerative processes (Amiri et al., 2024). This section introduces hydrogels for the management of periodontal soft tissue disease with respect to cutting-edge treatments for general chronic and diabetic periodontitis.

**FIGURE 2:**
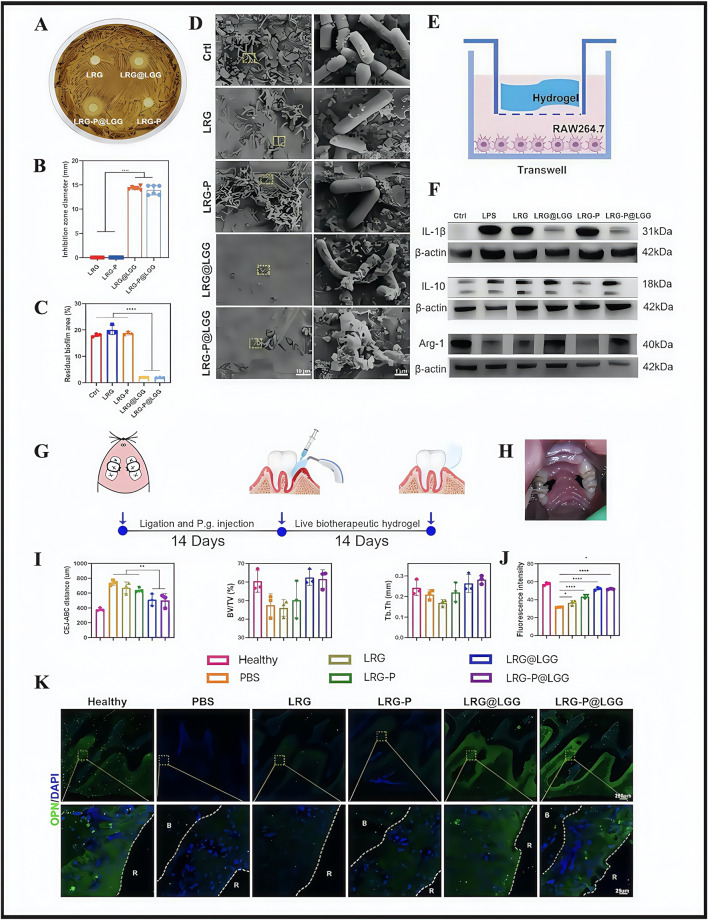
The effect of LRG-P@*LGG* on periodontitis in treatment-related experiments. **(A)** Inhibition zones and **(B)** quantification of diameters of hydrogels against *Porphyromonas gingivalis*. **(C)** The colony count (colony-forming unit; CFU) of *P. gingivalis* co-cultured with hydrogel cell-free supernatant (CFS). **(D)** Scanning electron microscope (SEM) images of *P. gingivalis* biofilms on hydroxyapatite tablets with different treatments. **(E)** Schematic of RAW264.7 cells co-cultured with hydrogels in the Transwell system. **(F)** Western blot analysis of interleukin (IL)-1β, IL-10, and Arg-1 protein expression levels in RAW264.7 cells. **(G,H)** Schematic of the experimental design and treatment process in rat periodontitis models. **(I)** Quantitative analyses of the cementoenamel junction–alveolar bone crest (CEJ-ABC) distance, the bone volume per tissue volume (BV/TV), and trabecular thickness (Tb.Th) of the alveolar bone detected by micro-CT. **(K)** Immunofluorescence images and **(J)** quantitative analysis of fluorescence intensity of osteopontin (OPN) (green) and DAPI (blue) in periodontal tissue. **(A–K)** (Li K et al., 2024) with permission from Elsevier.

According to the main clinical recommendations from the 2020 European Federation of Periology Clinical Practice Guidelines for the treatment of periodontitis (Sanz et al., 2020), current clinical treatments are based on basic periodontal and surgical approaches. Although these methods control plaque accumulation and thus periodontitis progression to some extent, they still face issues in term of incomplete plaque removal, unstable efficacy, abuse of anti-inflammatory drugs, high sensitivity to technology, and poor therapeutic experiences. Researchers, in the side-by-side comparison of traditional periodontal therapies with hydrogels, concluded that hydrogels had the following advantages: targeted delivery, ease of use, and minimally invasive (Das et al., 2025). From this, they constructed a multifunctional biotherapeutic hydrogel (LRG) equipped with *Lactobacillus rhamnosus GG* (*LGG*) for light responses, with the hydrogel (LRG-P@*LGG*) used for chairside periodontitis therapy (Li K. et al., 2024). The approach used active biotherapeutic products (LBP) in the periodontal mechanical treatment of incomplete plaque removal and reducing drug abuse problems. Current studies have also shown that *LGG* inhibits specific flora growth (Zhai et al., 2024). Thus, researchers screened *LGG*, which was shown to significantly inhibit the causative agent of periodontitis, *Porphyromonas gingivalis* (Reyes, 2021). LRG was designed by modifying cell-adhesion peptides and promoted tissue regeneration. In *in vitro* and *in vivo* evaluation studies, LRG-P@*LGG* showed good antimicrobial efficacy and effectively eliminated pre-existing bacterial films (Figures 2A–D). In terms of controlling inflammation, M1 macrophages release pro-inflammatory enzymes and cytokines that exacerbate alveolar bone loss in periodontitis, whereas M2 macrophages release anti-inflammatory cytokines that promote tissue recovery (Klopfleisch 2016). In a recent RAW264.7 cell study, LRG-P@*LGG*- and LRG@*LGG*-treated groups expressed significantly decreased M1 marker levels (interleukin (IL)-1β), while M2 marker (IL-10 and Arg-1) levels were promoted (Figures 2E,F). These observations suggested that LRG-P@*LGG* exerted potential immunomodulatory effects. In a rat periodontitis model, the LRG-P@*LGG*-treated group showed a particularly pronounced reduction in alveolar ridge bone resorption and bone regeneration in periodontal tissues (Figures 2G–K) Throughout the study, experimental rats showed no visible inflammation, necrosis or tissue injury signs in major organs, including the heart, liver, spleen, lungs, kidneys, and colon. Similarly, no noticeable flora variations were identified in oral and fecal flora from these rats, which suggested that LRG-P@*LGG* was biocompatible and did not adversely affect oral ecological balance in test rats. However, research gaps remain on whether interactions occur between *LGG* and hydrogels, predicting biodegradation rates, and resolving genetically engineered *LGG* issues with activity. Such issues must be overcome if these materials are to be used in clinical therapy.

Bacterial infections are initiating factors for periodontitis (Meyle and Chapple, 2015), but currently, subgingival anaerobes have been reported as highly resistant to conventional therapeutic agents (Sparbrod et al., 2022). Thus, applied wound dressings should be resistant to drug-resistant bacteria. In a recent study, researchers used catechol modification and nano-enzymes to enhance hydrogel properties and improve antioxidant and antimicrobial capabilities. The hydrogel dressing was formed using 3,4-dihydroxy-d-phenylalanine and combining polyvinyl alcohol with manganese dioxide nanoparticles (NPs) (PDMO hydrogel) (Hu et al., 2023). This approach alleviated the hypoxic, inflammation microenvironment by converting various free radicals (including total ROS-O_2_^-^·and OH·) to O_2_ via superoxide dismutase/catalase (Trivedi et al., 2015). The material also showed good antimicrobial effects against most subgingival anaerobes implicated in periodontitis. Moreover, the PDMO hydrogel can be used in combination with photodynamic therapy, with hydrogel photothermal properties showing good antimicrobial and antimicrobial film effects against *Staphylococcus aureus, P. gingivalis, and Escherichia coli* (inhibition rates were close to 100%) under 808 nm near-infrared (NIR) light. Finally, PDMO hydrogel biosafety was thoroughly investigated and showed good biocompatibility. Therefore, PDMO hydrogels adequately addressed subgingival anaerobic bacterial resistance in periodontitis. More so, as a local drug delivery system, the material had potential in clinically managing periodontitis and may undergo clinical translation in the future.

**FIGURE 3:**
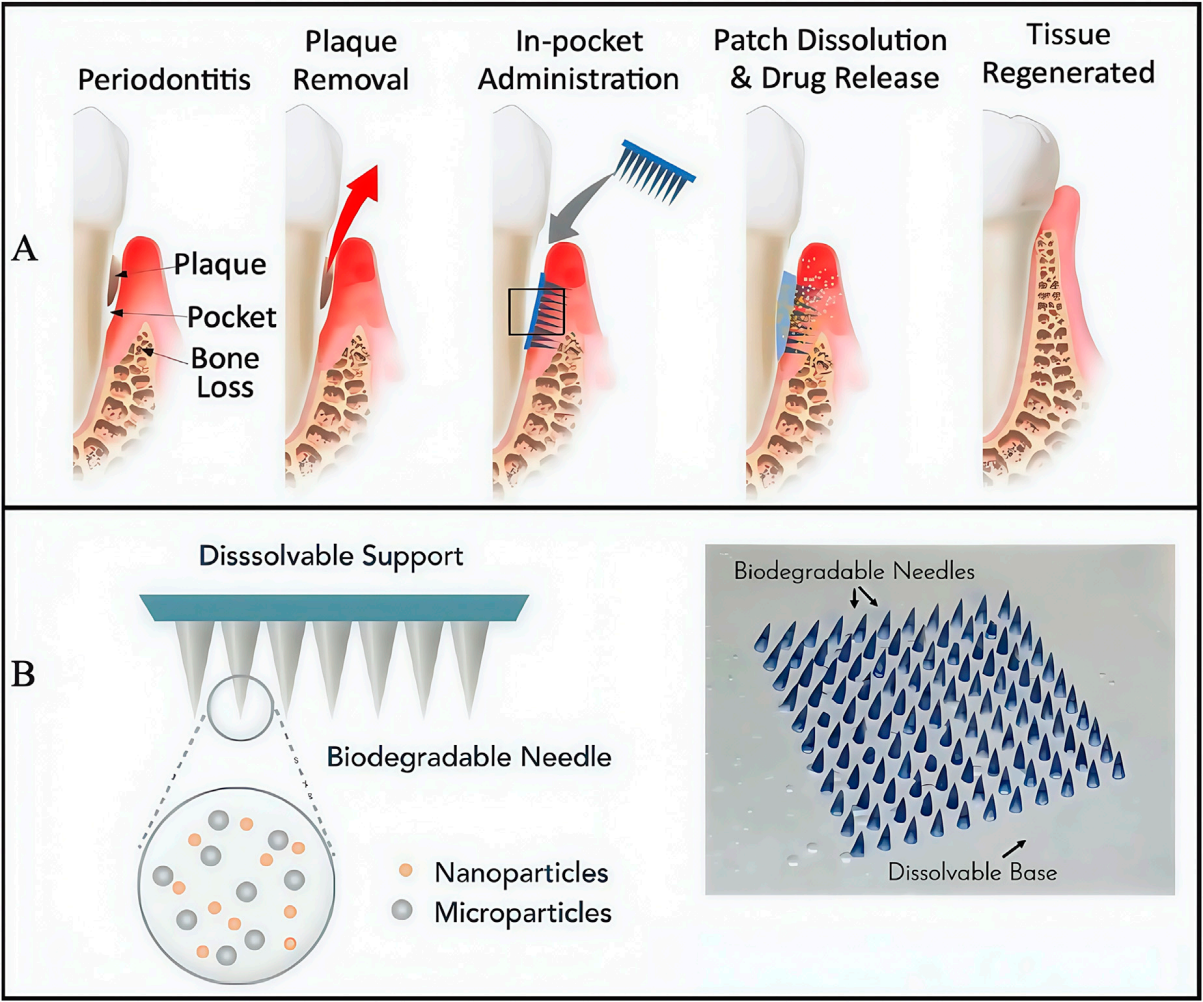
The structure of engineered immunotherapeutic periodontal methacrylated hydrogel microneedles (MNs) patch. **(A)** Planned approach for the immunotherapeutic periodontal MNs patch. **(B)** Overview of the design for the multifunctional MNs patch. **(A,B)** (Zhang et al., 2022) with permission from Matter.

In addition to innovating hydrogel dressings that carry drugs, researchers also modified hydrogel structures to generate a patterned microneedle (MN) patch. This approach subverted traditional injectable drug delivery routes by administering drugs via MN penetration into periodontal tissues (Figure 3A), thus ensuring drug delivery routes were more direct and effective. The approach consisted of a rapidly dissolving gelatin film that promptly released tetracycline, with biodegradable gelatine methacrylated (GelMA) methacrylated hydrogel MNs containing tetracycline-loaded poly (lactic-co-glycolic acid) NPs with cytokine-loaded silica microparticles facilitating sustained release (Figure 3B) (Zhang et al., 2022). Further MN degradation studies showed that half of MNs were degraded with collagenase in phosphate buffered saline (PBS) over 5 days, whereas MN degradation in human saliva was slightly slower and possibly accelerated degradation in the acidic environment at periodontitis disease sites. This issue has been an ongoing problem with GelMA-based hydrogels: their degradation cycle is not easily predictable and is highly influenced by the local microenvironment, particularly in acidic or high matrix metalloproteinase environments (Hou et al., 2025). Moreover, silica particle modification with heparin ensured cytokine loading and activity maintenance. When an MN patch comes into contact with periodontal tissue, basement membranes rapidly dissolve, immediately releasing large amounts of tetracycline, reducing bacterial concentrations in the periodontal pocket within a short period, and slowing down further disease development. Simultaneously, cytokines (with immune-regulating functions) penetrate tissues via MN’s to exert sustained and stable drug effects, thereby providing strong immune responses in diseased areas and avoiding irreversible long-term damage, such as bone resorption, tooth loss on periodontal tissues, and long-term damage (i.e., detachment). However, no clear biocompatibility evidence has been reported. In the future, if such materials are to be formally incepted into clinical settings, additional research is required on human biocompatibility profiles.

In comparing the aforementioned hydrogels, although all three demonstrated enhanced adhesion, their underlying mechanisms are different; PDMO hydrogels adopt chemical means, while MNs show improved hydrogel structures at physical levels to enhance adhesion. But, all three are more innovative in terms of the drugs carried by the hydrogel. LRG-P@*LGG* is improved by the addition of the active biological *LGG*, while in terms of drug tissue permeability, MNs are better and challenge existing drug transdermal absorption paradigms; hydrogel MNs puncture the mucosa so that drugs directly reach lesion areas. In terms of treatment modalities, the PDMO hydrogel and photodynamic therapy combination improves subgingival plaque inhibition, while LRG-P@*LGG* is used as an adjunct for bacterial inhibition after traditional mechanical treatment to reduce antibiotic abuse.

However, these hydrogel dressings lack clinical trial investigations, thus, more in-depth research is required so that these dressings can be used for clinical treatments, to provide better quality of care for patients.

Periodontitis is associated with several systemic diseases and conditions, including diabetes mellitus (DM), atherosclerosis, and rheumatoid arthritis (Hajishengallis and Chavakis, 2021; Konkel et al., 2019). Among these diseases, DM is a global epidemic characterized by a prolonged hyperglycemic state. Importantly, its bidirectional relationship with periodontitis has been extensively studied (Sun et al., 2022). Studies report that periodontitis occurs at higher rates and is more severe in patients with DM when compared to healthy individuals (Wang et al., 2023). In diabetics, high blood glucose levels often lead to increased reactive oxygen species (ROS) production and various pro-inflammatory cytokine release (Xiang et al., 2024). In particular, homeostatic imbalances between ROS levels and antioxidant defenses can also cause oxidative stress, which is implicated in periodontitis pathogenesis in diabetic patients (Karaca et al., 2022; Kocher et al., 2018). Therefore, antimicrobial, anti-inflammatory, and antioxidant therapies are required to treat diabetic periodontitis. Currently, antimicrobial agents (antibiotics or antimicrobials) are used as adjunct therapies that inhibit bacterial overgrowth. However, elevated antibiotic levels can cause oral pathogen resistance, leading to recurrent plaque overgrowth (Qi et al., 2024; Li et al., 2023b). Therefore, identifying more advanced therapeutic approaches is critical for diabetic periodontitis management. Importantly, hydrogel platforms are revolutionizing periodontitis management and have unique advantages: simple preparation and application, extracellular matrix-like networks, and tunable chemical modifications for multimodal therapy. These properties indicate that hydrogels are significantly superior to other local drug delivery systems (LDDS), including fibers, films, and NPs (An et al., 2024; Weiser and Saltzman, 2014).

**FIGURE 4:**
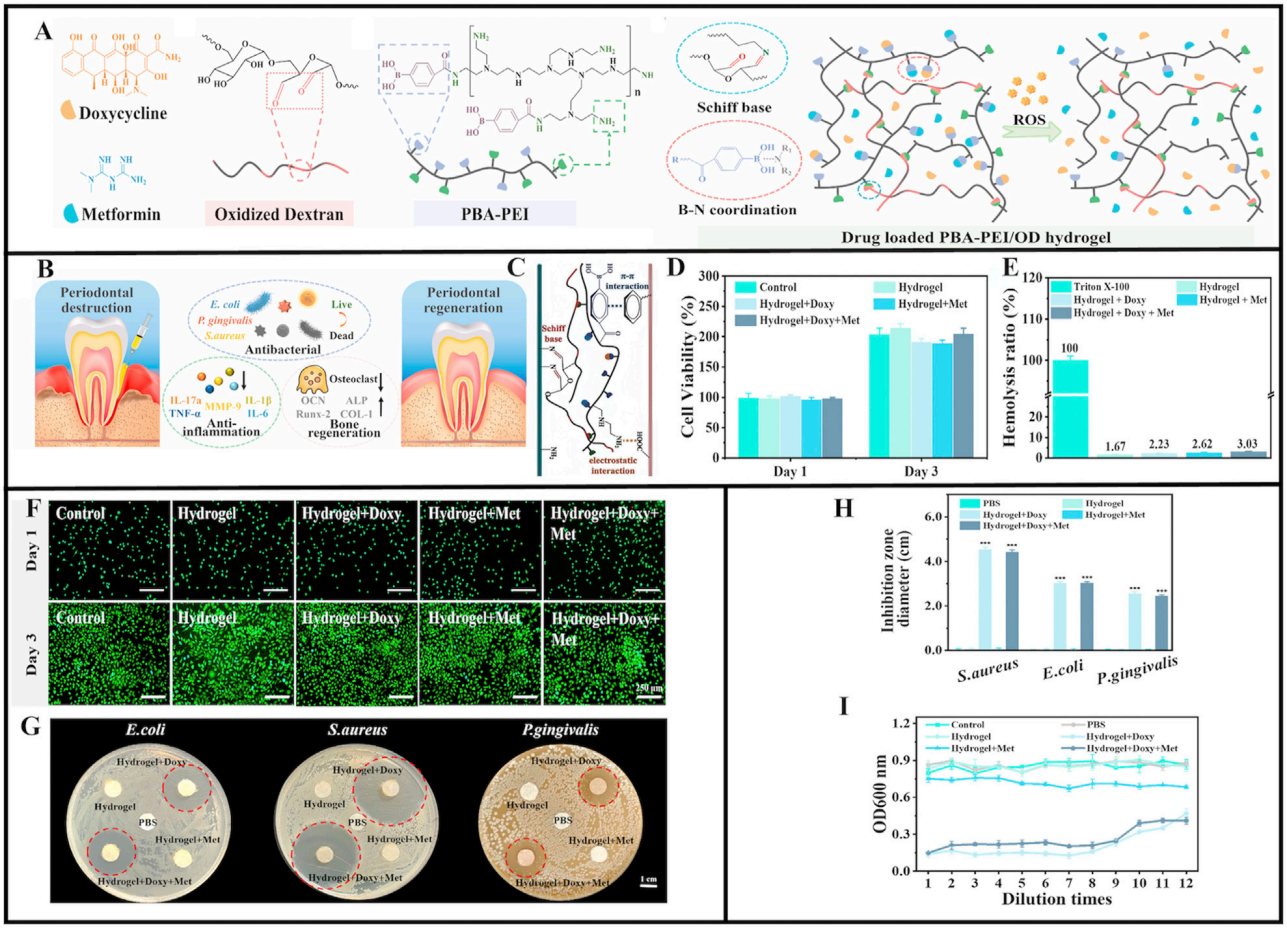
Schematic illustration of PBA-PEI/OD hydrogel. **(A)** The chemical structure of Doxycycline, Metformin, Oxidized dextran, and PBA-PEI. Scheme of the preparation process of PBA-PEI/OD hydrogel and its ROS-responsive drug delivery property. **(B)** Injectable Doxy + Met-loaded PBA-PEI/OD hydrogel exerts antibacterial effect and promotes periodontal regeneration. **(C)** The adhesion mechanism interpretation of the PBA-PEI/OD hydrogel. **(D)** Cell viability of L929 cells treated with hydrogel extracts for 1 and 3 days. **(E)** Hemolytic percentage of the hydrogels. **(F)** Live/Dead staining of L929 cells after incubated with the hydrogels extract for 1 and 3 days. **(G)** Digital images displaying representative agar disc diffusion test results for the hydrogels and PBS (negative control) against *Escherichia coli*, *Staphylococcus aureus*, and *Porphyromonas gingivalis*; the red dotted circles depict the zone of inhibition. **(H)** Inhibition zone diameter (n = 3) for the negative controls and the hydrogels. **(I)** Optical density (OD) values of *Porphyromonas gingivalis* inoculated in aliquots collected from the control group, PBS group and various hydrogel extracts after serial dilutions at 48 h. Values are presented as mean ± SD. **(A–I)** (Zhao X. et al., 2022) with permission from Elsevier.

In a previous study, oxidized dextran and phenylboronic acid-functionalized poly (ethyleneimine) (PBA-PEI) generated Schiff bases and formed injectable hydrogels (Figure 4A) (Zhao X. et al., 2022). Doxycycline (Doxy) and metformin (Met) were then added to the hydrogel to increase its glucose-lowering, antibacterial, and anti-inflammatory effects (Figure 4B) (Cummings and Torabinejad 2000; Cortes et al., 2018; Pereira et al., 2018; Araújo et al., 2017; Zhang et al., 2021). After injection into periodontal pockets, Schiff base production and π-π interactions between the hydrogel and tissue promoted hydrogel adhesion that withstood typical oral movements, and provided a basis for long-term hydrogel action in periodontal pockets (Figure 4C). The 3-(4,5-dimethyl-2-thiazoLyl)-2,5- diphenyl tetrazolium bromide (MTT) and Live/Dead staining assays showed good biocompatibility between drug-loaded PBA-PEI/OD hydrogels and original hydrogels (Figures 4D–F). *In vitro* drug release studies and accelerated Doxy and Met release from hydrogen peroxide (H_2_O_2_), the drug-loaded hydrogel showed adequate antibacterial efficacy against *S. aureus*, *E. coli,* and *P. gingivalis* (Figures 4G–I) (Zhao X. et al., 2022). The hydrogel was designed with B-N coordination and ROS responsiveness, ensuring high drug loading efficiency while also accounting for biosafety and on-demand degradation capabilities. This approach was unmatched by traditional treatment methods, and showed great potential for treating local diabetic periodontitis. However, the long-term biocompatibility effects of B-N coordination and boron accumulation risks require full verification. Also, matching ROS response thresholds to inflammation still requires further exploration, while drug synergistic effects and long-term efficacy data are needed.

**FIGURE 5:**
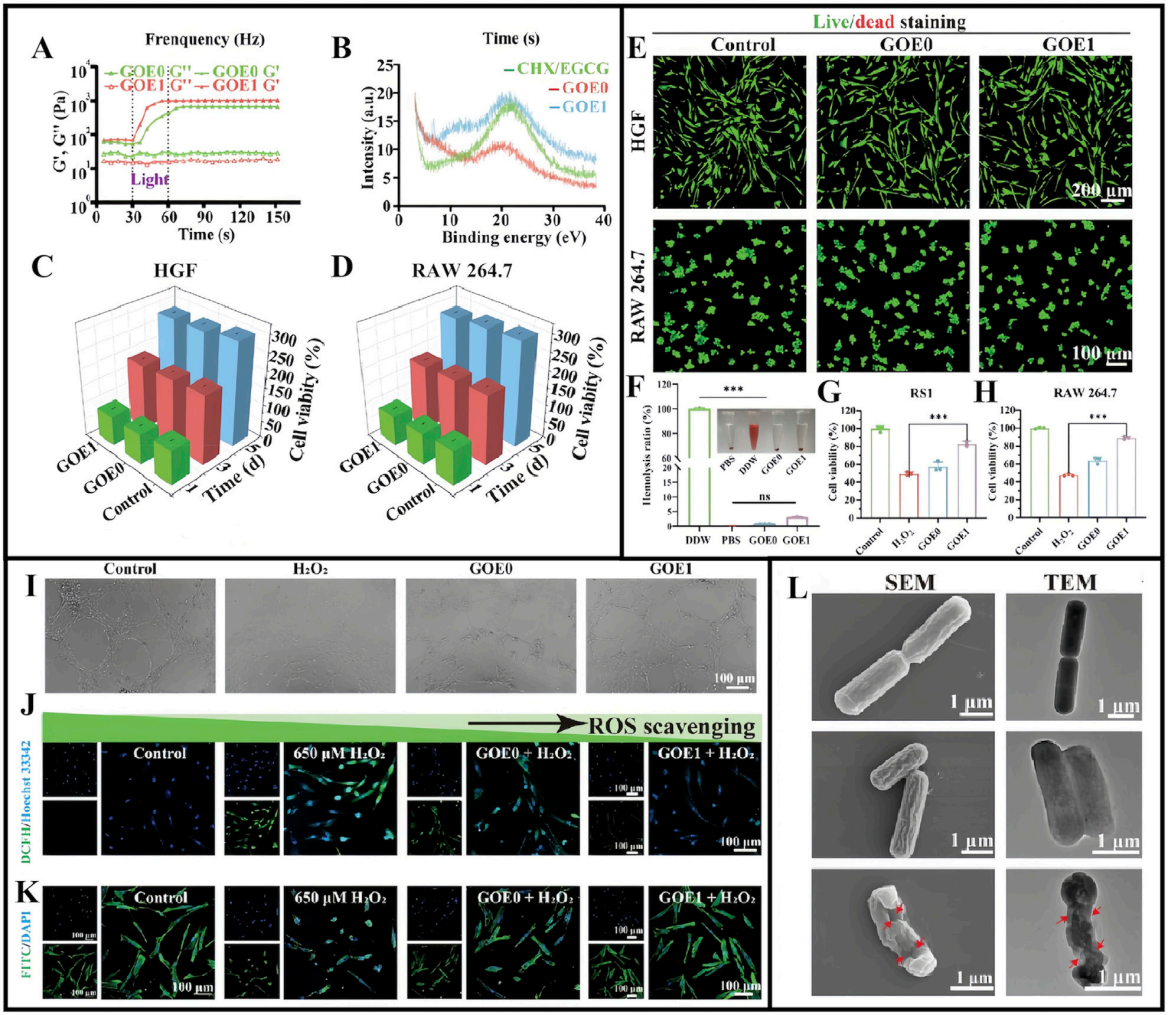
**(A)** Modulus of different hydrogels before and after illumination. **(B)** X-ray diffraction spectra for CHX/EGCG, GOE0, and GOE1. **(C,D)** The viability of HGF **(C)** and RAW 264.7 cells **(D)** assessed by CCK-8 assay in gel extract with full medium as control over 1, 3, and 5 days. **(E)** Live/dead staining for HGF and RAW 264.7 cells on day 3. **(F)** Blood compatibility assessed via a hemolysis test. **(G,H)** The ratio of survival for RS1 cell **(G)** and RAW 264.7 **(H)** cells in an H_2_O_2_-rich medium with varying hydrogels. **(I)** Assessment of tube formation in HUVECs under various treatments. **(J)** Confocal microscopy images and quantification of intracellular ROS levels. **(K)** F-actin staining depicting cell nuclei (blue) and cytoskeleton (green) on fibers. **(L)** SEM and TEM images displaying *Porphyromonas gingivalis* with marked cell membrane damage and degeneration. **(A–L)** (Ge et al., 2024) with permission from Wiley.

Hydrogel exploration for treating diabetic periodontitis is not limited to the aforementioned examples. For example, Ge et al. reported that chlorhexidine (CHX) and epigallocatechin-3-gallate (EGCG) could be encapsulated in hydrogels made from OHA and GelMA, resulting in a multifunctional controlled-release drug delivery system (GOE) with anti-ROS, antimicrobial, and anti-inflammatory effects. In their study, four different hydrogels were created with varying CHX/EGCG NP doses: 0, 0.5, 1, and 2 mg/mL and designated as GOE0, GOE0.5, GOE1, and GOE2, respectively (Ge et al., 2024). Gel mechanical properties were significantly enhanced after UV irradiation (Figure 5A), while powder X-ray diffraction analyses showed that CHX/EGCG incorporation changed the GOE crystal structure (Figure 5B) (Ge et al., 2024). GOE hydrogels had excellent hemocompatibility (<5% hemolysis) and excellent biocompatibility profiles at appropriate concentrations (GOE0, GOE1), making them suitable and promising materials for practical biomedical applications (Figures 5C–F). In free radical scavenging assays, RS1 cell and RAW 264.7 macrophage survival rates were significantly increased after hydrogel pre-treatment (Figures 5G,H). Additionally, GOE1-treated cells showed broader branching and greater total branch length, and indicated a GOE1 ability to support angiogenesis (Figure 5I). GOE1 incorporation into medium containing H_2_O_2_ effectively protected cells from oxidative stress in the DP microenvironment (Figures 5J,K). Thus, GOE1 was highly effective in protecting HGF from ROS-induced injury and creating a favorable normoxic environment, thereby supporting DP wound healing, reducing oxidative stress, and enhancing periodontal tissue regeneration (Ge et al., 2024). After GOE1 treatment, *P. gingivalis* levels were significantly reduced (reduced colony forming units) and demonstrated reduced cytoplasm levels, suggesting that GOE1 impeded bacterial regeneration by disrupting bacterial membrane integrity and improving antibacterial efficacy (Figure 5L) (Ge et al., 2024).

**FIGURE 6:**
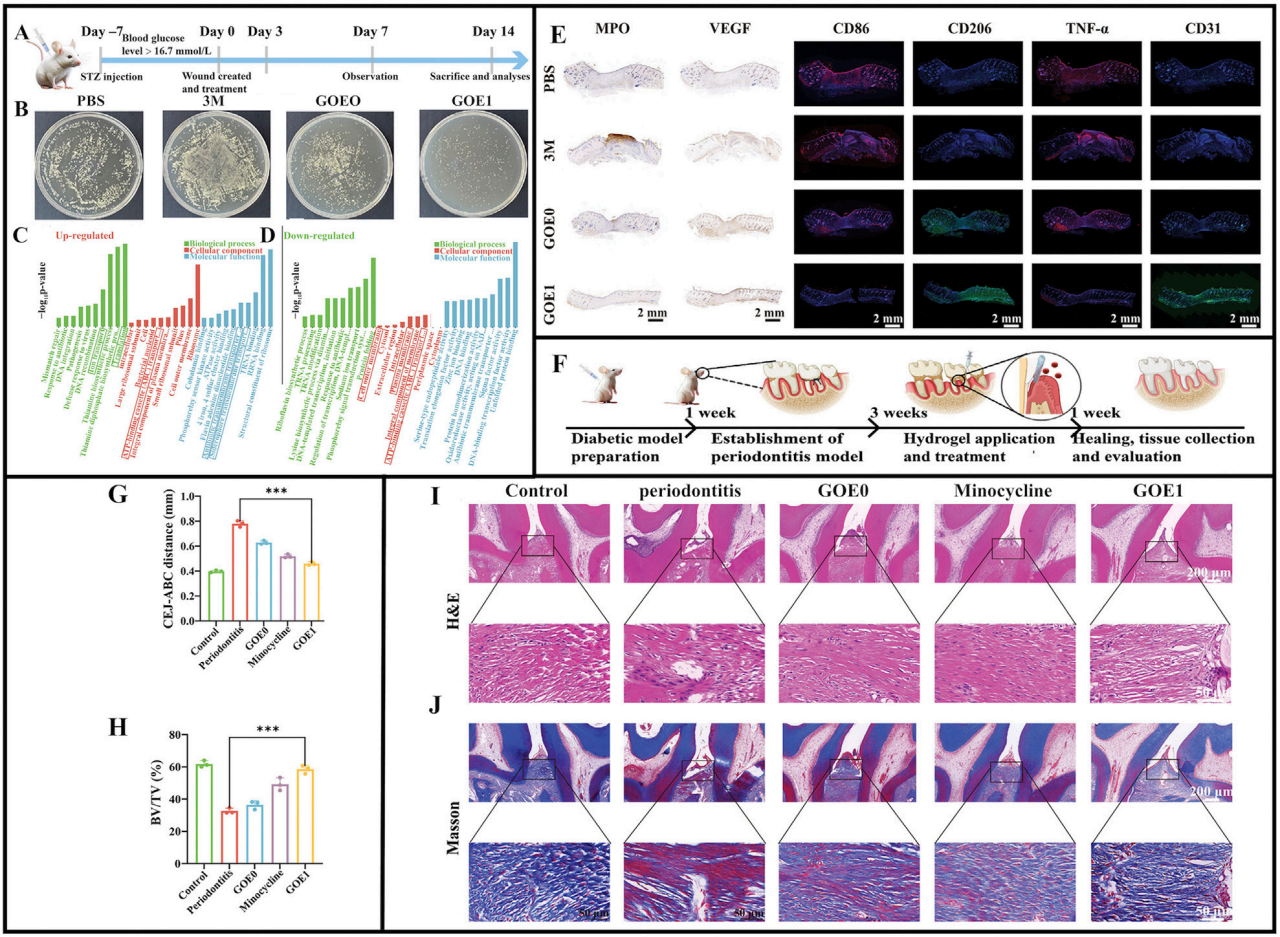
**(A)** Diagrammatic illustration of the timeline for wound healing. **(B)** Photographic evidence of *Staphylococcus aureus* colonies on TSB plates from various wound sites on day 21. **(C)** Outcomes of GO analysis for proteins that are upregulated. **(D)** Outcomes of the GO analysis for proteins that are downregulated. **(E)** Typical immunofluorescence images showing CD86, CD206, TNF-α, and CD31, along with immunohistochemical staining for MPO and VEGF in various treatment groups after 14 days. **(F)** Diagrammatic representation of periodontitis induction using a ligature. **(G)** Evaluation of the distance from the CEJ to the ABC. **(H)** BV/TV: bone volume relative to total volume. **(I,J)** Rat periodontal tissue sections prepared for H&E staining and Masson’s trichrome staining. **(A–J)** (Ge et al., 2024) with permission from Wiley.

In wound healing studies on infected rats (Figure 6A), GOE1 significantly enhanced wound healing *in vivo* by promoting neoepithelial formation and increasing collagen accumulation, representing better antimicrobial properties than other hydrogels (Figure 6B) and showing excellent hemostatic capacity (Figures 6C,D) and promoting microvascular regeneration (Figure 6E) (Ge et al., 2024). The study induced periodontitis in rats using ligature wires (Figure 6F). The CHX/EGCG hydrogel-treated group had significantly lower periodontitis data (Figures 6G,H). Hematoxylin & Eosin and Masson staining showed that elastic and collagen fibers in bone tissue were denser and more ordered under GOE1 gel actions, similar to normal tissue structures (Figures 6I,J). GOE1 significantly reduced inflammatory cell infiltration in gingival tissue, thus, GOE1 hydrogels significantly enhanced bone preservation. Finally, the GOE hydrogel treatment group had lower TNF-α, CD86, and MPO levels, and the inflammatory milieu was significantly improved, demonstrating that BOE hydrogels significantly reduced periodontal inflammation (Ge et al., 2024). Furthermore, the hydrogel achieved precise and quantitative drug release via the oxidative cleavage of non-covalent bonds. This significantly improved uncontrollable drug release issues in traditional periodontitis treatments, enabling drugs to exert long-term effective actions toward periodontal tissue and thereby enhancing treatment outcomes. In terms of biocompatibility, standard blood tests and histological data from multiple key organs indicated that GOE1 showed no obvious cytotoxicity, confirming excellent biosafety and biocompatibility profiles. Further research should examine the molecular mechanisms whereby EGCG enhances antimicrobial effects and quantitatively analyze dose-dependent CHX and EGCG synergistic effects.

**FIGURE 7:**
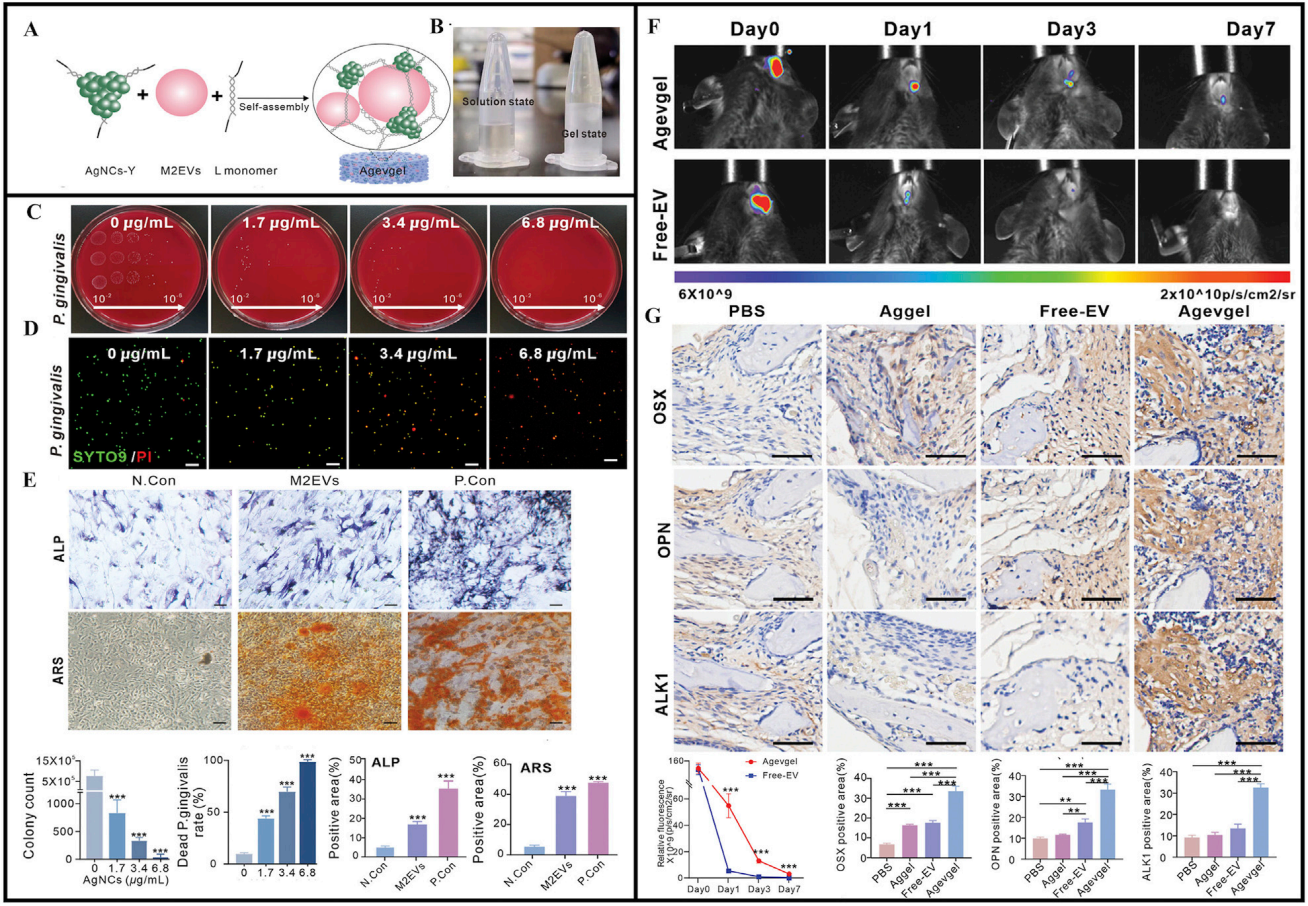
**(A)** Schematic of the synthesis and structure of Agevgel. **(B)** Agevgel in the solution and the gel states. **(C)** Flat colony images and quantitative counts of *Porphyromonas gingivalis* at different dilutions (10^2^–10^6^) treated with AgNCs. **(D)** Fluorescent images and quantitative analysis of the live/dead staining of *Porphyromonas gingivalis* with SYTO9 (green)/PI (red) after treatment with AgNCs. **(E)** ALP and ARS staining images and quantitative analysis after the induction of BMSCs with M2EVs on days 7 and 14, respectively. **(F)** *In vivo* fluorescence and quantitative analyses of the relative fluorescence changes at the DABD areas on days 0, 3, and 7 after treatment with Agevgel or free M2EVs. M2EVs are stained by PKH26. **(G)** Representative IHC staining images and quantitative analysis of OSX, OPN, and ALK1 expression levels at the defect sites of diabetic mice in the PBS, EV, Aggel, and Agevgel groups on day 21. **(A–G)** (Peng et al., 2024) with permission from Wiley.

Another study developed a multifunctional DNA-based hydrogel (Agevgel) by integrating silver nanoclusters (AgNCs) and M2 macrophage-derived extracellular vesicles (M2EVs) (Figure 7A). The preparation showed antimicrobial, anti-inflammatory, osteogenic, and immunomodulatory properties in promoting the reconstitution of diabetic alveolar bone defects (DABD) (Figures 7B–D) (Peng et al., 2024). Agevgel’s functionality was mainly derived from AgNC and M2EV (Kang et al., 2020; Chen et al., 2022; Zhang and Lo, 2022; Soma et al., 2022). This study suggested that M2EVs not only promoted osteogenic differentiation and mineral deposition, but also regulated osteogenic differentiation (Figure 7E) (Peng et al., 2024). In contrast, Agevgel sufficiently prolonged M2EV release *in vivo* to significantly reduce administration frequencies (Peng et al., 2024). Moreover, Agevgel promoted DABD reconstruction by stimulating proliferation and osteogenesis (Figures 7F,G) (Peng et al., 2024). Thus, Agevgel enhanced DABD reconstruction via sustained M2EV release and antibacterial AgNC actions. It synergistically ameliorated inflammation, enhanced proliferation, induced osteogenesis, and prevented bacterial infection in the long term, and showed great potential in promoting DABD reconstruction. Agevgel’s ability to significantly accelerate alveolar bone damage healing in diabetic patients may effectively improve alveolar bone repair in future clinical treatments, thereby addressing the limitations of traditional bone repair materials in terms of their narrow application range and limited efficacy (Peng et al., 2024). Agevgel showed multiple functions, including antibacterial, anti-inflammatory, and tissue regeneration properties, which were precisely regulated via ROS response release. Importantly, its long-term sustained-release characteristics reduced its administration frequency rates, and combined with its non-invasive delivery method, it effectively improved patient compliance. When compared to traditional mechanical therapy combined with antibiotic treatments, Agevgel demonstrated superior efficacy with fewer side effects, markedly reducing patient discomfort and technical sensitivity. Data from mouse liver and kidney analyses also indicated that Agevgel showed good biocompatibility and safety profiles. Thus, *in vivo* DNA framework stability and long-term AgNC toxicity should be further evaluated, which may contribute to manufacturing standards and the commercial development of this hydrogel.

**FIGURE 8:**
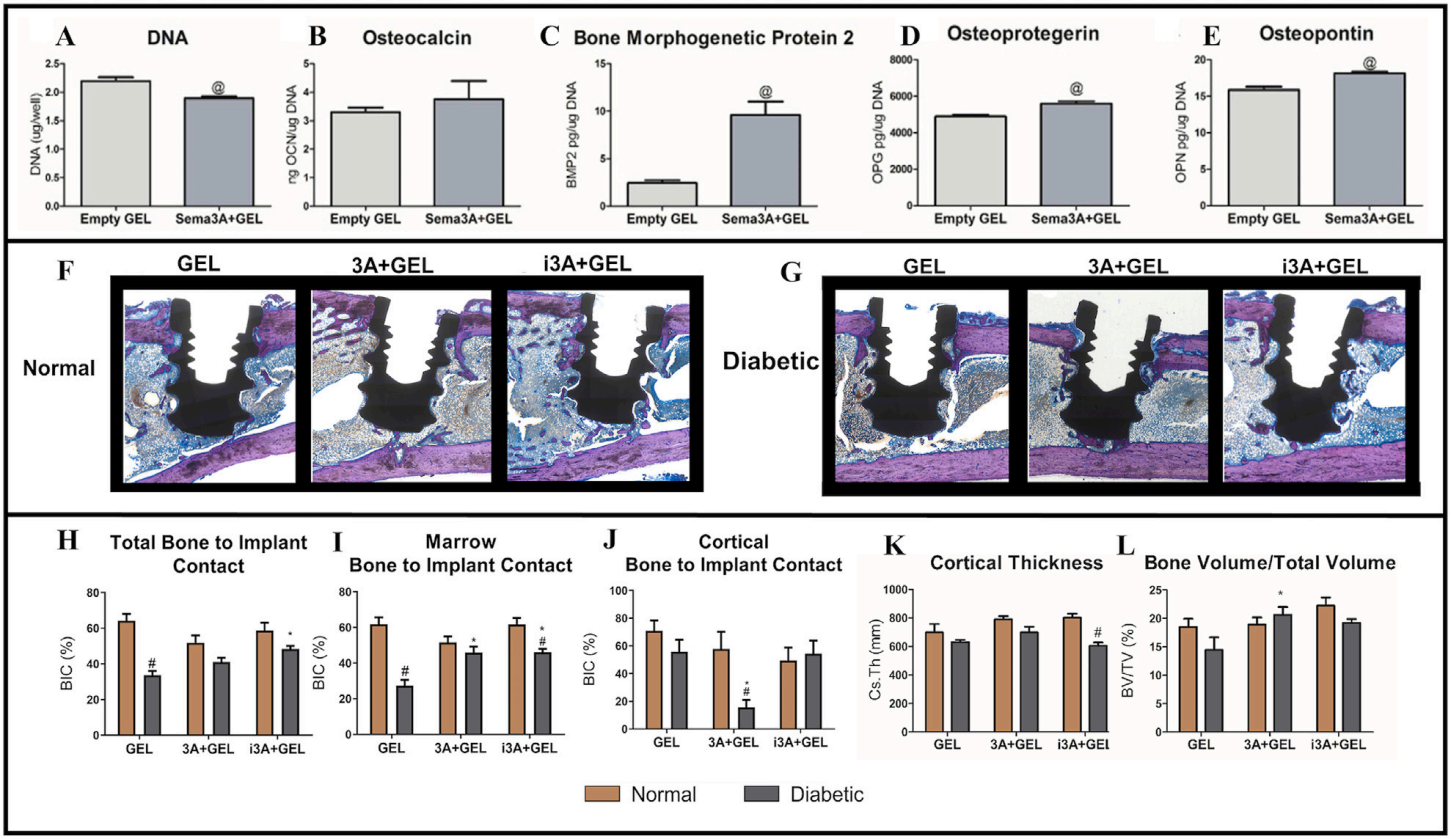
The comparison to empty hydrogel was assessed by the **(A)** DNA content, **(B)** osteocalcin production, **(C)** bone morphogenetic protein two production, **(D)** osteoprotegerin, **(E)** and osteopontin. **(F–G)** All sections were stained with Stevenel’s Blue/van Gieson stain and coverslipped. Osteoid was stained purple and connective tissue was stained blue. **(H)** Total Bone to Implant Contact was analyzed further into two subregions: **(I)** Marrow Bone to Implant Contact and **(J)** Cortical Bone to Implant Contact. In addition, **(K)** Cortical Thickness, and **(L)** Bone Volume/Total Volume were quantified. **(A–L)** (Deng et al., 2023) with permission from Acta Biomater.

Dental implants can improve patient wellbeing and health by restoring missing teeth. However, poor bone quality associated with conditions such as Type 2 DM and osteoporosis can affect implant survival rates (Wild et al., 2004; Deepa et al., 2015). Sema3A is a known osteoprotegerin that increases bone formation and inhibits bone resorption (Verlinden et al., 2016; Chen et al., 2017; Negishi-Koga and Takayanagi, 2012; Kim and Koh, 2019; Hayashi et al., 2012). In previous research, Sema3A promoted osteoblastic differentiation in bone marrow mesenchymal stem cells (BMSCs) from diabetic rats and attenuated the inhibitory effects of high glucose levels on osteogenesis (Qiao et al., 2018; Zhang et al., 2019). Deng et al. reported bone regenerative effects for Sema3A using Sema3A click hydrogels (Deng et al., 2023). BMP2, OPN, and OPG levels were increased in cells treated with Sema3A+ GEL medium when compared to cells treated with empty GEL medium, demonstrating that click hydrogels retained Sema3A biological activity (Figures 8A–E) (Deng et al., 2023). When compared to local injections, Sema3A delivery (doped into click hydrogels) also increased total BIC (Figures 8F–L) (Deng et al., 2023). However, for clinical treatments, local injections were more difficult to perform, while patients experienced more physical and psychological pain. In contrast, hydrogels may be more advantageous as non-invasive drug delivery carriers in reducing patient discomfort during treatment. Click hydrogels also do not swell as much under physiological conditions when compared with other hydrogels, which is associated with improved mechanical properties, reduced slippage from application sites, and reduced local nerve compression (Deng et al., 2023). This suggests that these hydrogels can improve implant placement success rates in diabetic patients (Deng et al., 2023). The innovation behind this hydrogel lies in its combination with Sema3A bone-protective functions and the sustained-release properties of the hydrogel, thereby overcoming traditional injection limitations with non-invasive delivery approaches. Future studies should explore the interaction mechanisms between Sema3A and the pathological microenvironment in diabetes (e.g., hyperglycemia and oxidative stress), which may highlight material failure risks.

In other research, an injectable temperature-sensitive hydrogel system, based on chitosan/sodium β-glycerophosphate, was developed (Liu D. et al., 2025). Through multifunctional modification, the system’s ability to respond to complex pathological environments was significantly enhanced. The approach integrated photothermal antibacterial and immune-modulating functions, enabling a multidimensional intervention for peri-implantitis, and in turn, addressing antibiotic resistance or insufficient efficacy issues associated with traditional non-surgical treatments.

Experimental results showed that the hydrogel system rapidly heated up to over 48°C under 808 nm NIR light irradiation, effectively killing *S. aureus* and *P. gingivalis*. It achieved over 50% cumulative drug release within 7 days, and its antibacterial efficacy was unaffected by light exposure duration. *In vitro* cell studies also confirmed excellent biocompatibility. Furthermore, sustained simvastatin release significantly promoted CD4^+^ T cell differentiation to regulatory T cells and upregulated Foxp3, TGF-β, and IL-10 expression, thereby inhibiting secretion of the pro-inflammatory factor IL-17. Animal studies further confirmed that this hydrogel, in a diabetic mouse implant-associated periodontitis model, not only reduced periodontal pathogens by 1.5 orders of magnitude, but also effectively promoted bone regeneration by regulating the local immune microenvironment.

This hydrogel system overcame traditional treatments that relied solely on antibacterial agents or passive repair. It provided a non-surgical, low-resistance treatment strategy for diabetes-related implant complications and represents the application of multifunctional biomaterials in chronic inflammatory diseases. However, underlying immune regulation and antibacterial action mechanisms remain unclear, while material production processes are relatively complex and require further improvements. These areas should be examined in future research and development studies (Liu D. et al., 2025).

### 4.2 Treating periodontal hard tissue diseases

Alveolar bone defects caused by wounds or infection in oral and maxillofacial regions are very common in clinical practice. These diseases usually affect patients’ daily functions, such as diet, occlusion, and speech (Siow et al., 2023). Currently, the main treatment option for alveolar bone defects is bone grafting; the gold standard is autologous bone grafting (Sheikh et al., 2017). Traditional therapies face challenges such as limited donor bone sources, complications at bone graft sites, and potential immune rejection, which prevent the effective treatment of these bone defects (Moeintaghavi et al., 2007). When compared to traditional growth factors and cell-based therapies, hydrogels have superior quality controllability and are better sourced. As 3D crosslinked polymer networks, hydrogels have unique molecular architectures that efficiently retain substantial amounts of water, so they provide ideal microenvironments for cellular growth and tissue formation. Notably, their tunable design enables the precise matching of geometric and mechanical properties at damaged tissues. This ensures optimal integration while minimizing mechanical disruption to surrounding structures. Furthermore, through precise chemical modulation, hydrogels closely mimic the biocompatibility and mechanical behaviors of native tissues. They significantly enhance tissue integration efficiency and mitigate risks such as immune rejection and adverse reactions. Thanks to these exceptional properties, pro-angiogenic hydrogels have demonstrated unparalleled potential in alveolar bone regeneration applications (Pan J. et al., 2020). Hydrogels are 3D crosslinked polymer network structures that can hold large amounts of water in their interlocking molecular networks. At present, various multifunctional hydrogel systems have been widely used to treat alveolar bone diseases. The research has mainly focused on hydrogel multifunctionality and stimulus responsiveness, and also hydrogels with multifunctional properties, such as self-repairing, antimicrobial, and angiogenesis promotion qualities (Yu et al., 2024; Wu S. Y. et al., 2023; Pan J. et al., 2020). Some researchers have also focused on hydrogel pH responsiveness, temperature responsiveness, and other stimuli-responsive properties and explored their application in alveolar bone repair (Wang W. et al., 2023).

**FIGURE 9:**
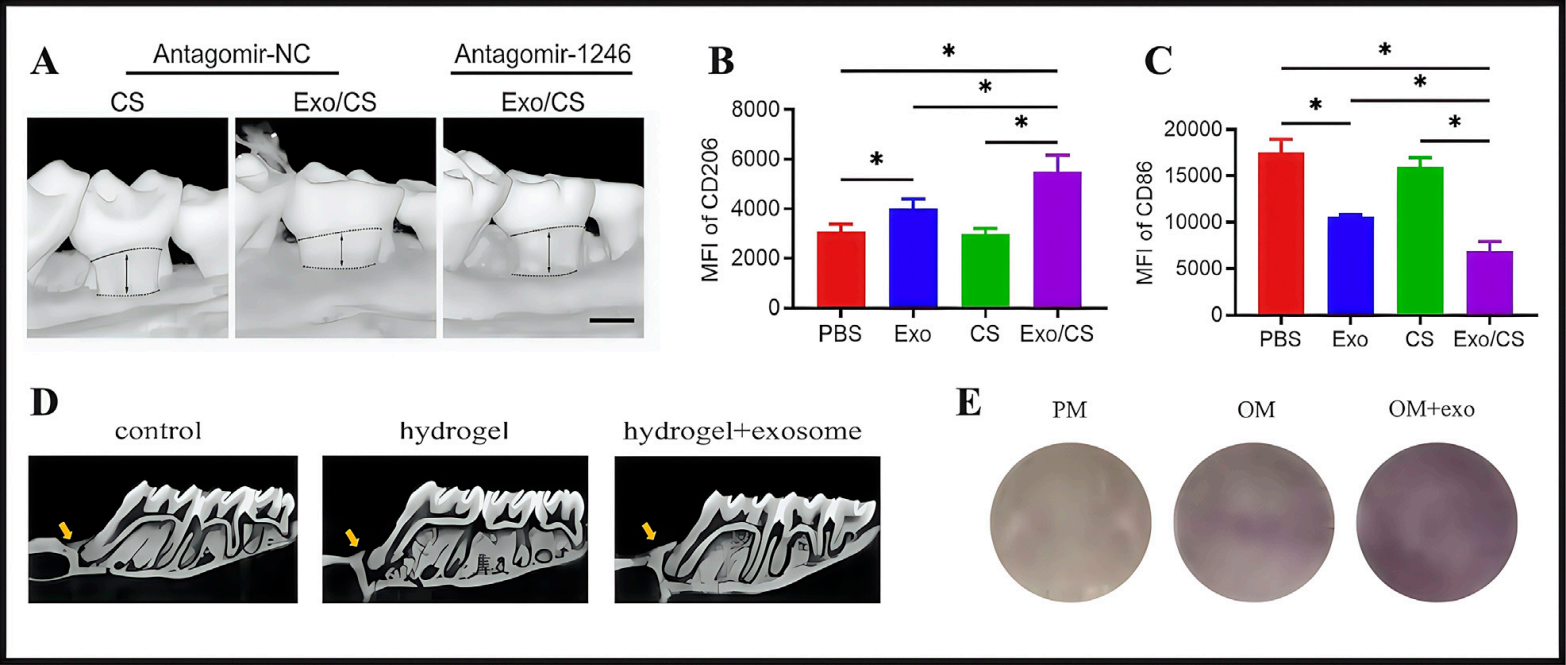
**(A)** The maxillary alveolar bone surrounding the area was imaged using micro-CT 4 weeks after DPSC-Exo/CS treatment. **(B)** Analysis of flow cytometry statistics showing the MFI of CD206^+^ cells in the CD11b^+^F4/80^+^ cell population in the periodontium across different groups of P mice (n = 6 per group). **(C)** Flow cytometry data analysis focusing on the MFI of CD86^+^ cells in the CD11b^+^F4/80^+^ cell population within the periodontium of each group of P mice (n = 6 per group). **(D)** Alveolar bone micro-CT images taken 4 weeks following PDLSCs-Exos treatment. **(E)** After 14 days of osteogenic induction, ALP staining was performed. **(A–C)** (Shen et al., 2020). **(D,E)** (Zhao Y. et al., 2022) with permission from Shen, Z and Zhao, Y.

Previous studies also confirmed the therapeutic potential of exosomes in promoting alveolar bone defect repair (Chew et al., 2019). Hydrogels have garnered significant research interest as carriers in extending the lifespan of exosomes and enhancing their effectiveness. Shen and colleagues used a hydrogel system containing exosomes derived from dental pulp stem cells (DPSCs) to regenerate alveolar bone in mice with periodontitis. The authors developed a DPSC-Exo-incorporated chitosan hydrogel (DPSC-Exo/CS) by combining DPSC-derived exosomes (DPSC-Exo) with a chitosan hydrogel (CS). By facilitating pro-inflammatory macrophage polarization toward an anti-inflammatory phenotype and inhibiting bleeding in defective alveolar bone, the material accelerated alveolar bone and periodontal epithelium healing in mice with periodontitis (P mice) and helped repair alveolar bone (Figure 9A). In studies, experimental P mice treated with PBS-, CS-, and DPSC-Exo were compared. CD206 (anti-inflammatory marker) expression was significantly increased while CD86 expression (pro-inflammatory marker) was significantly decreased in P mice treated with DPSC-Exo/CS (Figures 9B,C). Thus, DPSC-Exo/CS promoted the conversion from a pro-inflammatory to an anti-inflammatory phenotype in periodontal tissues in P mice, which potentially alleviated experimental periodontitis and reduced alveolar bone loss (Shen et al., 2020). Building on these findings, periodontal ligament stem cells (PDLSCs) exhibited significant advantages over alternative stem cell sources, particularly in terms of tissue availability, accessibility, and robust regenerative capacity. Zhao and Lo (2022) isolated exosomes from PDLSCs and combined them with a Gelatin-Sodium Alginate Hydrogel (Gel-Alg Hydrogel) to evaluate their osteogenic potential in an alveolar bone defect rat model. Gel-Alg Hydrogel cytotoxicity was assessed (CCK-8 proliferation assays), which revealed no apparent cytotoxic effects. The authors also evaluated different exosome concentration effects on BMSC proliferation and osteogenic differentiation. Studies also confirmed exosome effects on osteogenic differentiation *in vitro* using alkaline phosphatase staining. *In vitro* studies revealed that PDLSC-derived exosomes (PDLSC-Exos) effectively stimulated BMSC proliferation and induced osteogenic differentiation (Figures 9D,E).

Shen et al. used DPSC-Exo/CS encapsulated in a CS hydrogel to treat periodontitis via macrophage phenotype switching. In contrast, Zhao et al. investigated PDLSC-Exo delivery via an Gel-Alg Hydrogel in promoting alveolar bone regeneration. Although both studies used stem cell-derived exosomes and hydrogel carriers, fundamental distinctions were observed. The core discrepancy related to the divergent nature of the disease models and therapeutic objectives. Shen targeted inflammatory periodontitis, necessitating the concurrent resolution of inflammation and bone regeneration. Conversely, Zhao used a pure bone defect repair model, placing a greater emphasis on osteogenic differentiation. This divergence led to differences in disease model selection: Shen used a ligature-induced periodontitis model, while Zhao created a surgical mechanical bone defect model. Their mechanistic emphases also differed significantly: Shen highlighted immunomodulation via M1–M2 macrophage polarization, whereas Zhao concentrated on direct osteoinduction via BMSC proliferation and differentiation. Thus, the divergent properties of both hydrogel systems were apparent. In Shen’s study, the CS hydrogel served a dual function as a sustained-release vehicle and an immunomodulatory agent. In contrast, Zhao’s Gel-Alg Hydrogel primarily functioned as a physical scaffold. The former’s thermosensitive nature facilitated injectable delivery, while the latter’s requirement for calcium-mediated crosslinking potentially compromised clinical maneuverability. In terms of methodological rigor, Shen demonstrated greater comprehensiveness, incorporating four control groups, establishing in vitro–in vivo mechanistic continuity, and functionally validating key microRNAs (miRNAs). Zhao’s study presented significant methodological constraints, with a no exosome-only treatment group, a lack of validation for critical miRNAs, and potential artifactual signals from lipophilic dye (DiD) labeling. Notably, while hydrogel-alone groups were ineffective in both studies, reported exosome monotherapy efficacy was incongruent. Shen reported that DPSC exosomes alone, though less potent than the composite, exhibited significant therapeutic effects. Zhao, however, omitted an exosome-only group. This discrepancy potentially arose from several factors: the requirement for a scaffold to maintain defect space in Zhao’s bone model, the potentially shorter half-life of PDLSC exosomes, or differences in administration protocols. Future research should prioritize therapeutic efficacy comparisons of exosomes from different cellular sources in chronic inflammatory models, and develop engineered exosome strategies incorporating targeting modifications. For clinical translation purposes, DPSC-derived exosomes may be more suitable for inflammatory control, whereas PDLSC-derived exosomes may have enhanced regenerative potential in scenarios requiring pure bone regeneration.

**FIGURE 10:**
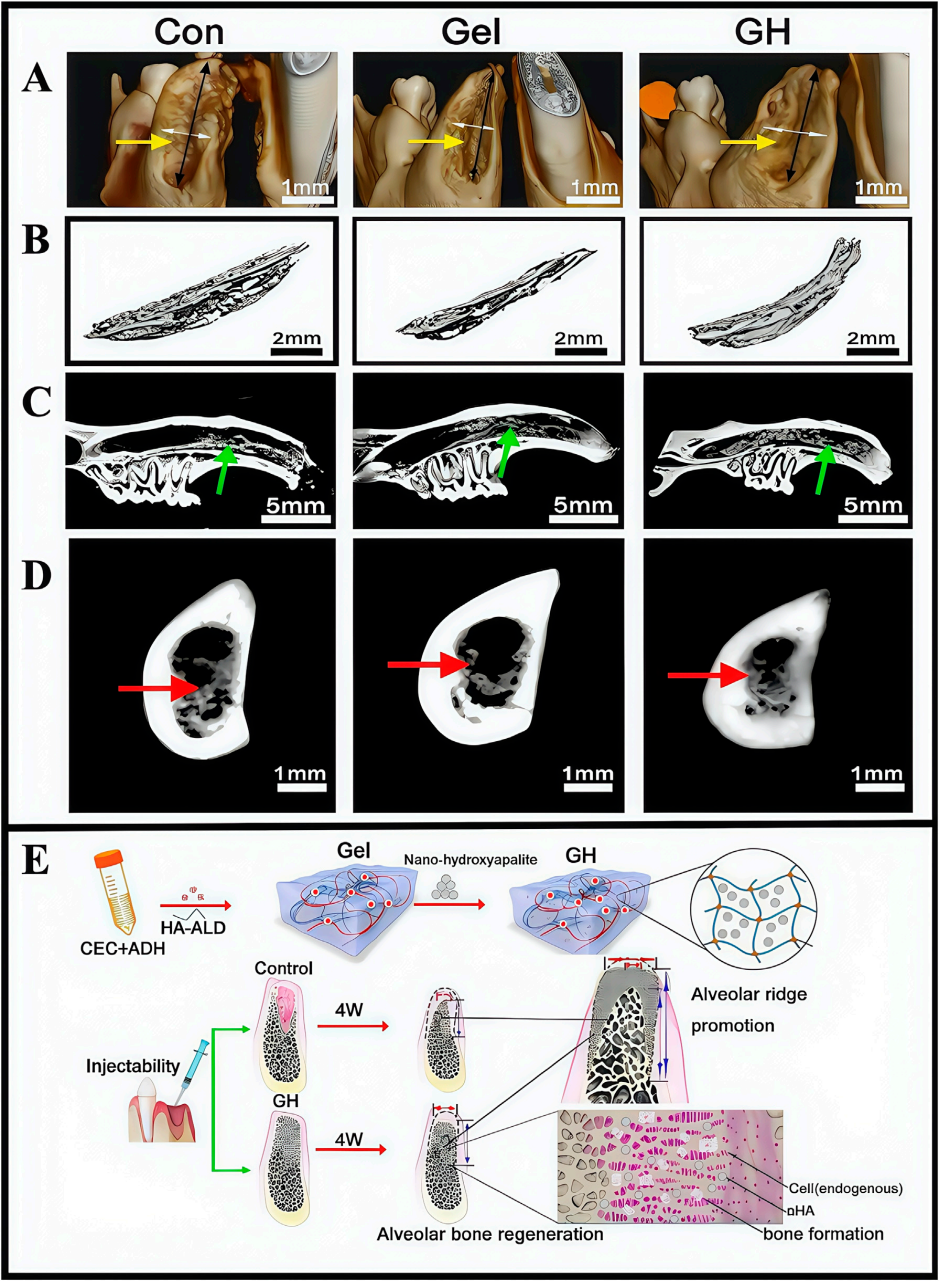
**(A)** Three-dimensional reconstruction of tooth extraction. **(B)** New bone tissue in the tooth extraction. **(C)** Analysis of the lateral sagittal section of the extraction. **(D)** Analysis of the coronal section of the extraction side. **(E)** Schematic illustration showing the fabrication process of hydrogel incorporating nano-hydroxyapatite. **(A–E)** (Pan Y. et al., 2020) with permission from Elsevier.

Hydrogels, in addition to the components they carry, should also be investigated in terms of material selection and optimization. Conventional polymer hydrogels demonstrate poor cell orientation and adhesion properties, and lack proteins required for osteoblast function so they cannot directly bind to host bone wells (Curvello et al., 2019). Nano-hydroxyapatite is not only similar to bone in terms of chemical composition and crystal structure, but is the main inorganic component of bone. It supports osteoblast adhesion and migration in host bones, has bone conduction and induction properties, and has been used for oral bone tissue regeneration (Nie et al., 2019). Pan et al. developed a scaffold composite containing inorganic-organic materials that were optimized for nano-hydroxyapatite and soft self-repairing hydrogel (i.e., hydrogel-hydroxyapatite, GH) scaffolds. *In vitro* degradation studies demonstrated that the hydrogel underwent 50% self-degradation within 2 weeks and complete degradation within 4 weeks. Histopathological evaluations revealed no significant inflammatory responses. Furthermore, the self-healing hydrogel consisted mainly of biocompatible polysaccharides, a natural biomaterial class that showed excellent biocompatibility (CCK-8 assays). In studies, an alveolar bone defect model of mandibular central incisor in eyeball traction rats was established, and then the scaffold was minimally invasively extracted. Finally, the material was shown to have extraordinary alveolar ridge preservation abilities, which has provided new ideas for repairing clinical bone defects (Figure 10) (Pan Y. et al., 2020). Although several studies have reported that hydrogels have significant potential in treating alveolar bone defects, clinical application obstacles remain. These limitations are mainly manifested as clinical operation difficulties and a lack of comprehensive safety assessments. Therefore, more basic and clinical studies are required to advance clinical hydrogel applications in treating alveolar bone defects.

Recent clinical trials and meta-analyses also validated hydrogel efficacy for alveolar bone regeneration. A 2024 randomized controlled trial demonstrated that injectable hydrogels loaded with bone morphogenetic protein-2 (BMP-2) (osteoinductive agent) and mesenchymal stem cell-derived exosomes significantly enhanced bone volume and density in patients with periodontal bone defects when compared to traditional bone grafts (Xu et al., 2024). These advancements underscored the potential of hydrogels in addressing autologous graft limitations, such as donor site morbidity and unpredictable resorption rates. Future research should optimize hydrogel degradation kinetics and facilitate large-scale multicenter trials to standardize clinical protocols.

### 4.3 Treating oral mucosal diseases

The oral mucosa consists of stratified squamous epithelium and underlying connective tissue. Oral mucosal diseases, which are a broad category encompassing various lesions affecting the oral mucosa and related soft tissues, exhibit significant abnormalities in both epithelial and lamina propria layers. In certain cases, these abnormalities may extend into deeper anatomical structures, such as submucosal connective tissue, minor salivary gland tissue, and muscular tissue. Erythema, erythroleukoplakia, leukoplakia, submucosal fibrosis, and lichen planus are consistent and diverse manifestations associated with these diseases. Currently, the standard management approach for these conditions involves administering local pharmacological therapies.

In clinical practice, the highly dynamic and diversified nature of bacterial communities in the oral cavity environment represents substantial challenges to drug efficacy and accessibility. Thus, researchers are actively investigating new materials. Hydrogels have garnered considerable attention due to their distinctive biological properties, such as adhesiveness, long-term sustained drug release, and outstanding capacity as drug delivery carriers. In subsequent discussions, we comprehensively explore the latest research advancements regarding hydrogel applications in treating oral mucosal diseases.

#### 4.3.1 Treating OLP

OLP is a chronic T-cell-mediated inflammatory disease affecting the oral mucosa. The condition is represented by intermittent episodes and is categorized as two main forms: erosive and non-erosive. Patients with OLP commonly experience mucosa roughness and burning sensations. Exposure to spicy foods, high temperatures, or other irritants exacerbate these symptoms, leading to heightened sensitivity and burning pains at affected sites. Although much research has focused on OLP etiology, its precise pathogenesis remains undetermined. Current therapeutic approaches primarily focus on symptomatic relief, aiming to reduce inflammation, prevent infections, and alleviate pain through topical medication. Corticosteroids are widely acknowledged as preferred OLP treatments. Nevertheless, prolonged topical use poses risks, including the potential induction of oral candidiasis and adverse effects such as mucosal atrophy. Moreover, the unique characteristics of the oral cavity, such as limited mucosal surface areas, restricted drug-carrying capacity, and rapid drug loss due to frequent salivary rinsing, often result in suboptimal therapeutic outcomes (Chan et al., 2000; Hamour et al., 2020; Lodi et al., 2020; Louisy et al., 2024). Previously, researchers developed mucosal adhesion delivery systems to overcome limitations associated with oral mucosal medication (Fratila et al., 2024). These systems encompassed bioadhesive particles, mucosal adhesive buccal tablets, and polymeric mucosal adhesive membranes, among others. Despite significant advances, the complex and moist oral environment poses considerable challenges in terms of the adhesion and sustained drug release of therapeutic materials for oral mucosal diseases. Moreover, previous material studies have focused on the oral mucosa in general rather than specific oral diseases (Liu J. et al., 2025). In light of this, with widespread hydrogel dressing applications in biomedicine, many researchers have examined hydrogel dressings for treating OLP. One such treatment is a viscous hydrogel dressing (Dental Tough Adhesive, DenTAl), which exhibited robust mechanical properties, strong adhesion to various moist and dynamically moving oral tissues, and potentially prolonged drug release. This biomimetically tough adhesive dressing comprised two layers: an adhesive surface and a dissipative matrix. Unlike conventional adhesives, the tough adhesive used a multi-component adhesion strategy to generate strong adhesion to underlying moist tissues. When compared to *in situ* polymerized hydrogel adhesives and bandages, the toughness and adhesive energy of the matrix was enhanced by several orders of magnitude. This research indicated that hydrogel dressings could effectively adapt to moist oral cavity environments, conform to OLP lesions, and facilitate prolonged active anti-inflammatory drug release, thereby improving therapeutic outcomes (Wu D. T. et al., 2023). Despite such breakthroughs in adhesive performance and sustained-release duration shown by DenTAI, several critical limitations persist. Therapeutically, its efficacy remains unverified in OLP animal models, casting uncertainty on its actual anti-inflammatory effects and adhesive properties in pathological mucosa. In terms of long-term biosafety, the absence of a degradable hydrogel design raises concerns about potential safety risks from prolonged mucosal adhesion. Furthermore, comprehensive assessments of local/systemic side effects during extended drug release periods are lacking. Critically, biocompatibility evaluations were conducted over short durations and were insufficient to validate safety profiles under chronic exposure conditions. Pharmacokinetically, the system failed to achieve precise spatiotemporal control over drug release. Despite these limitations, DenTAl has shown superior material properties for the oral environment, highlighting hydrogel dressings for OLP treatment. Future research should prioritize biological safety profiles, precise drug release mechanisms, drug degradation rates, and the establishment of more animal studies confirming the therapeutic effects of hydrogel-loaded drugs for OLP.

As described, the most commonly used OLP treatment drugs are steroids, which have specific clinical application limitations, such as poor patient compliance and short drug residence times. To address this, an innovative adhesive Poloxam spray hydrogel was designed that released hydroxypropyl-β-cyclodextrin (HP-β-CD) in combination with dexamethasone for OLP treatment (Diaz-Salmeron et al., 2021). HP-β-CD improved dexamethasone solubility in hydrogels, while Poloxam was temperature-sensitive and facilitated the conversion of hydrogel sol and gel forms. Poloxamer hydrogels, recognized for their non-irritating properties toward oral mucosa, are widely used as hydrogel systems; however, suboptimal adhesion properties are a limitation. To address this challenge, researchers strategically incorporated sodium alginate and xanthan gum (functional modifiers) to enhance mucoadhesive performance while maintaining biocompatibility. This composite formulation demonstrated synergistic interfacial interactions via hydrogen bonding and electrostatic forces between polysaccharide chains and mucosal glycoproteins, thereby achieving sustained adhesion without compromising the inherent non-toxic characteristics of the base hydrogel matrix. In other studies, Huangyuan gum prolonged hydrogel retention times on oral mucosa to the greatest extent. The hydrogel, in a spray preparation with good drug release and adhesion capabilities, improved dexamethasone retention times, efficacy, and patient compliance (Diaz-Salmeron et al., 2021). Consequently, this hydrogel fabrication strategy provided a novel methodology for preparing spray formulations carrying certain water-insoluble drugs. However, no animal studies were conducted to verify hydrogel effects in real oral environments, and later experimental supplements are needed to further prove hydrogel efficacy.

Current OLP therapies face challenges such as rapid drug loss and poor therapeutic efficacy. To address these issues, most hydrogel systems have focused on enhancing mucoadhesive properties and achieving sustained drug release in the oral cavity. However, limitations remain, including a lack of relevant *in vivo* studies validating actual drug-loaded hydrogel efficacy in the oral environment, as well as insufficient research examining long-term hydrogel biocompatibility and intraoral degradation rates. In future research, these clinical limitations should be addressed to expand the practical applicability of these hydrogels.

#### 4.3.2 Treating OSF

**FIGURE 11:**
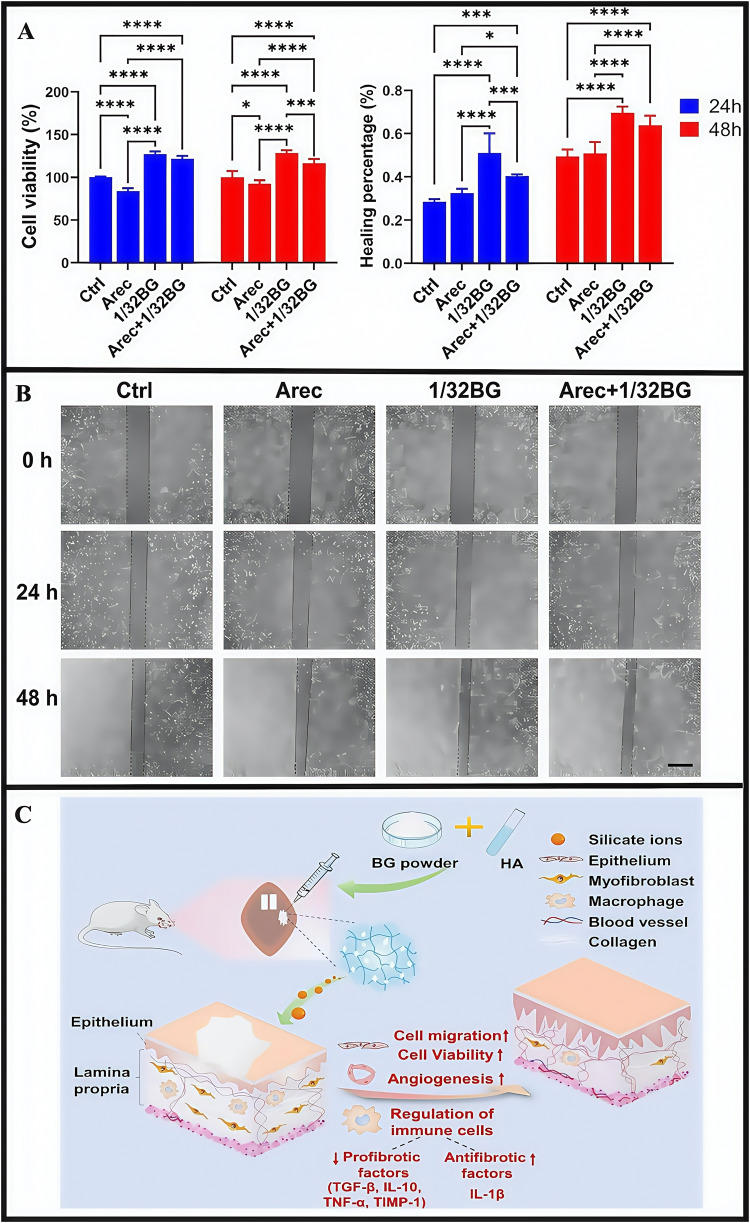
**(A)** Cell viability of epithelial cell after stimulation with Arec and/or 1/32BG for 24 and 48 h **(B)** Cell migration of epithelial cells after stimulation with Arec and/or 1/32BG for 24 and 48 h. Scale bar = 500 μm. **(C)** The mechanism hypothesis of BG/HA in the treatment of OSF. **(A–C)** (Guo et al., 2023) with permission from Elsevier.

OSF is a chronic, inflammatory and potentially malignant oral mucosal disease; it is characterized by abnormal collagen deposition and classified as a potentially oral malignant disorder (Zhi et al., 2024; Sharma et al., 2020). Basic OSF pathological mechanisms involve imbalanced collagen synthesis and degradation. The main clinical symptoms include burning sensations in the oral cavity when eating spicy food, and a series of symptoms that affect oral function, specifically manifested as limited mouth opening, difficulties in opening the mouth, dry mouth, and swallowing disorders. Chewing areca nut constitutes a significant risk factor for the development of OSF. Arecoline, the main areca nut component, acts on oral fibroblasts, promoting collagen synthesis, damaging the oral mucosa, triggering inflammatory responses, and simultaneously damaging endothelial cells. This leads to a series of pathological changes in patients with OSF, including reduced blood vessels and ultimately resulting in OSF development (Shih et al., 2019; Shen et al., 2020). Currently, clinical treatments mainly include: selective surgery, drug therapy, and mouth-opening exercises (Jones et al., 2024; Qin et al., 2023). Surgical treatment is selective and has strict indications; it is invasive and only suitable for patients who do not respond to other treatments and need to increase their mouth opening. However, surgery may lead to scar hyperplasia, thereby limiting mouth opening. Drug treatment cycles are long and follow-up frequencies are high, thereby affecting the quality of life for patients. Early drug treatments involve corticosteroids, which can relieve some symptoms, but the reversal of fibrous deposition effects is weak. Long-term corticosteroid use can exert significant side effects and disease recurrence risks are high after drug withdrawal. Oral opening practice is merely an adjunctive treatment and cannot fundamentally solve the problem. Recently, laser treatments reported good prospects and may effectively improve oral opening conditions in patients (Gupta and Jawanda 2021). However, due to certain medical staff requirements and associated high costs, its development has limitations. Therefore, no effective treatments exist for OSF in clinical practice, thus quality of life and prognosis outcomes for patients are poor. Currently, some drugs target a single pathological process, with limited therapeutic effects. If a more effective treatment is needed, multiple pathological processes must be targeted. Therefore, more studies are required to identify therapeutic methods that could limit multiple pathological processes and effectively improve OSF clinical efficacy. Currently, many drugs have shown excellent pharmacological effects and few side effects, but limitations remain for their clinical use. For instance, some drugs may cause mechanical damage to the oral mucosa and show rapid dissolution and instability. Importantly, hydrogel dressings have brought some hope to resolving the clinical use of these drugs. Researchers reported that bioglass (bioinorganic material) promoted wound healing and angiogenesis. It was also used to treat diabetic wounds and improve pulmonary fibrosis, but applications for oral settings remain understudied (Li Y. et al., 2024; Chen. et al., 2021). To combat this, researchers developed an injectable sodium hyaluronate/45S5 bioglass composite hydrogel (BG/HA), which continuously released silicate ions (Guo et al., 2023), while encapsulation with hyaluronidase reduced potential bioactive glass damage to the oral mucosa. BG/HA enhanced epithelial cell activity and migratory capacity, thereby repairing damaged oral mucosal tissue in OSF (Figure 11) (Huang et al., 2022; Hoppe et al., 2011; Zhai et al., 2012; Wu et al., 2022). In other research, silicate ions promoted angiogenesis (provide sufficient nutrition for the oral mucosa), accelerated collagen metabolism while inhibiting excessive deposition, and inhibited inflammatory macrophage expression levels to help regulate the OSF immune microenvironment (Huang et al., 2018). In animal studies, BG/HA significantly alleviated mucosal pallor and restricted mouth opening in rats with OSF, and was readily metabolized *in vivo* without accumulation, with no significant toxic side effects. From CCK-8 proliferation assays, cells cultured with the hydrogel exhibited favorable proliferative activity at both 24 h and 48 h, demonstrating good biocompatibility. Compared with mice receiving BG/HA treatment, it was found that mice receiving glucocorticoid treatment had more significant weight loss, indicating that the side effects of BG/HA were significantly fewer. In animal studies, the subcutaneous injection of the hydrogel (10 mL) into rat dorsal regions showed the almost complete disappearance of the hydrogel from the back skin at 48 h post-injection. Similarly, *in vitro* degradation studies demonstrated consistent results: all hydrogel groups exhibited significant mass losses exceeding 80% after 48 h of degradation. Both studies demonstrated that BG/HA possessed excellent degradation properties.

**FIGURE 12:**
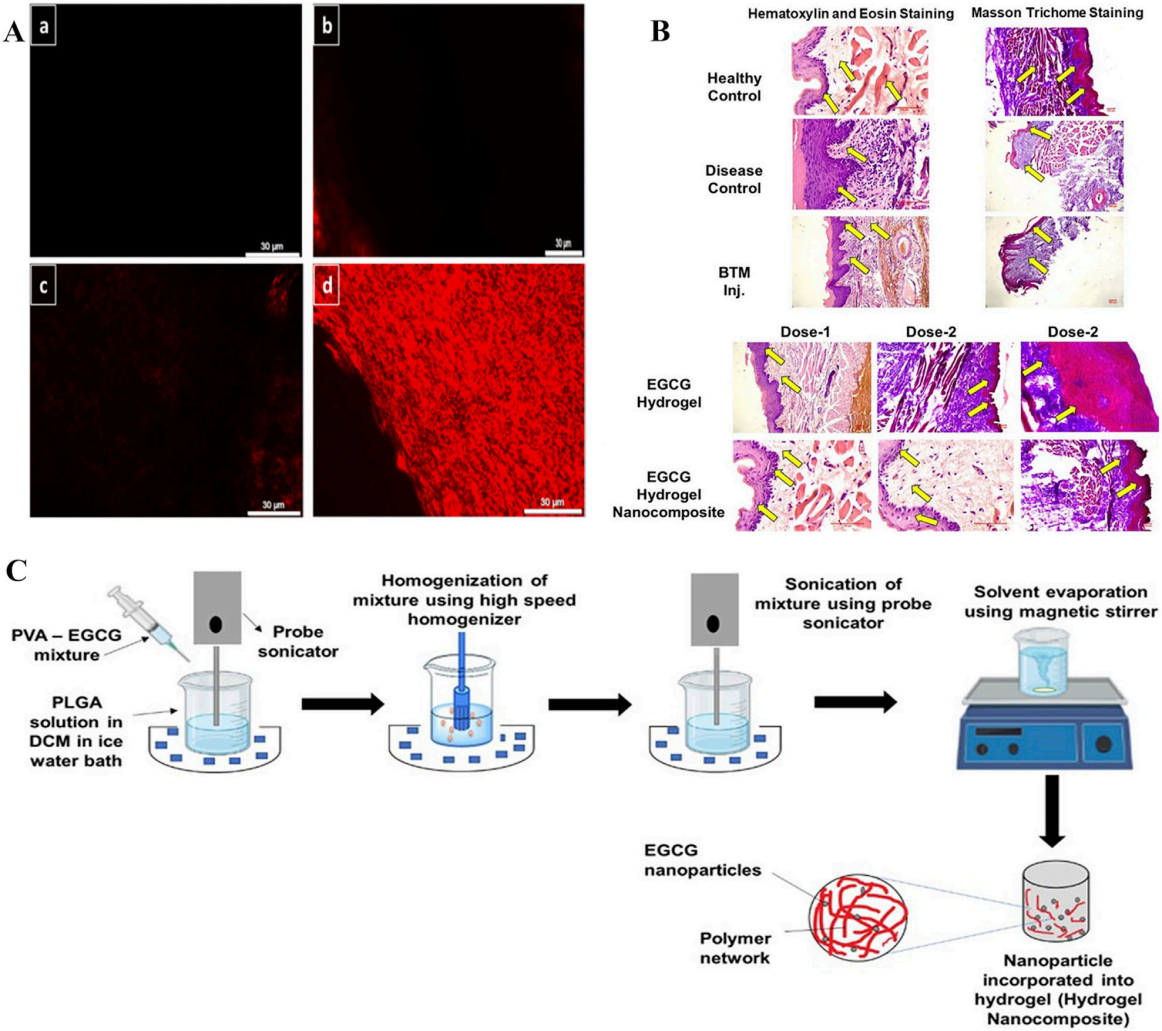
**(A)** Modified EGCG-SA NanoCubes demonstrate superior penetration: a. Control buccal mucosa, b. Rhodamine solution-treated buccal mucosa, c. Fluorescent-tagged unmodified NanoCubes treated buccal mucosa, and d. Fluorescent-tagged modified NanoCubes treated buccal mucosa. **(B)** Comparison of histopathological images between BTM injection and EGCG hydrogel. **(C)** EGCG-PLGA nanoparticle preparation and hydrogel nanocomposite (HNC) formulation. **(A)** (Mehta et al., 2021) **(B,C)** (Mehta et al., 2024) with permission from Elsevier and Mehta, C. H.

The main EGCG catechin in green tea has antioxidant, antibacterial, anti-ionizing radiation, and beneficial cardiovascular effects. When compared with other polyphenols such as quercetin, EGCG had higher biological safety profiles. Previous research indicated that EGCG attenuated collagen deposition by suppressing the TGF-β/SMAD signaling axis, which induces fibroblast-to-myofibroblast transition thereby promoting fibrogenesis (Lin et al., 2020). However, EGCG is a hydrophilic material that cannot easily penetrate the lipophilic oral mucosa, has poor transdermal properties, and poor stability and low bioavailability characteristics (Sahadevan et al., 2023; Alam et al., 2022). Therefore, methods are required to improve EGCG permeability and stability toward the oral mucosa. From these observations, researchers proposed using EGCG hydrogel dressings to treat OSF (Mehta et al., 2023). EGCG exerted multi-target effects, significantly downregulating collagen mRNA expression (types 1A2 and 3A1) and TGF-β1. Consequently, EGCG reduced collagen synthesis and TGF-β1 concentrations more effectively than traditional betamethasone (BTM) injection therapy, markedly improving mouth opening and ameliorating OSF in rat models. The study indicated that the hydrogel carrying EGCG was important in OSF pathological processes. However, no detailed research was conducted on the hydrogel, and more emphasis was placed on explaining EGCG pharmacological effects. No research was conducted on material mechanical properties, adhesiveness, and drug release abilities. In other work, researchers examined hydrogel material development, where nanocubes (NanoCubes) loaded with EGCG modified by stearylamine (SA) and integrated into adhesive hydrogels (NanoCubogel) were developed for local OSF treatment (Mehta et al., 2021). NanoCubogel (lipid cubic phase hydrogel) was composed of curved, continuous lipid bilayers, separated by a water channel network. The lipids that formed the cubic phase were relatively insoluble in water, so they could be used in oral environments. The material withstood considerable biological shear forces and could not easily fall off the oral mucosa, thereby providing conditions for effective drug release (Barriga et al., 2019). By modifying hydrogel dressing surfaces with SA (to make it positively charged), NanoCube penetrance into buccal mucosa layers was enhanced, and its biosafety improved (Jadav et al., 2024). When compared with unmodified hydrogel dressings and pure drugs, using NanoCubogel to carry drugs appeared to resolve EGCG limitations in clinical use, and significantly improved EGCG permeability, controllability, and sustained release in the oral mucosa (Figure 12A). However, researchers did not conduct *in vivo* studies in their OSF mouse model to verify drug effectiveness. Also, certain difficulties have been encountered in preparing lipid NP hydrogel dressings. Studies have reported that polymer nano-hydrogels are more stable and easier to prepare. Therefore, researchers generated EGCG-encapsulated NP-loaded adhesive hydrogel nanocomposites (HNCs) for OSF treatment (Figure 12C) (Mehta et al., 2024). During preparation processes, biodegradable PLGA NPs, which are widely used in drug delivery systems, were used to prepare HNCs. HNCs are a class of nanomaterials that integrate nanoscale properties with hydrogel matrices, while exhibiting excellent biocompatibility and showing prolonged mucosal retention and sustained drug release capabilities. These characteristics mean that HNCs are suitable for advanced drug delivery systems and tissue engineering applications (Amiri et al., 2021). The hydrogel dressing was modified with octadecylamine to a positively charged surface. This modification enhanced hydrogel dressing permeability on oral mucosa and contributed to better EGCG penetration into oral tissues (Salatin et al., 2016). Furthermore, as a carrier, internal octadecylamine particle polymer layers formed tight barriers, which helped control EGCG release and enable its continuous and stable release into target tissues. In *in vivo* studies, the EGCG-loaded NP group significantly improved opening conditions. Experimental data showed that collagen and TGF-β1 levels were significantly decreased, antioxidant levels increased, and epidermal layer thickness was close to that of the normal mucosa (Figure 12B). Thus, NPs carrying EGCG may effectively improve the disease state by regulating collagen and TGF-β1 levels. In *in vivo* studies, both visual observations and histopathological examinations (microscopy) revealed no significant alterations in mucosal tissues or parenchymal organs in mice injected with the HNC formulation when compared to BTM injection or EGCG hydrogel groups, indicating that HNCs exhibited favorable biocompatibility. This study added animal experiments for verification based on NanoCubogel, and obtained the effectiveness verification at the molecular level and histopathological level in the mouse model.

From these observations, the strategies using hydrogel dressings to treat OSF are basically the same; they exert effects on OSF pathological processes such as collagen deposition. Hydrogel dressings exhibit favorable mechanical properties (e.g., adhesive strength), enabling their utility in prolonging drug retention within dynamic oral environments where conventional formulations face challenges in long-term maintenance. At the same time, hydrogel dressings with unique 3D structures can be used to load some drugs with therapeutic potential in order to solve clinical use limitations, such as EGCG instability and degradation, and also alleviate possible adverse effects of bioactive glass. Thus, researchers can accelerate novel therapeutic development for clinical management and potentially reverse OSF pathological changes. However, these aforementioned studies mostly focused on research and development related to traditional hydrogels, but hydrogels are also represented by stimulus-responsive types, such as temperature-sensitive, pH-responsive, and ion-responsive types. By analyzing these types and the microenvironment characteristics of different oral mucosal wounds, more targeted hydrogels can be developed. While hydrogel dressings theoretically enable long-term localized drug delivery–thereby alleviating procedure-associated pain from repeated injections in conventional therapies to enhance patient compliance and therapeutic outcomes–existing research on hydrogel biomaterials critically lacks empirical clinical validation of actual patient efficacy feedback. Future studies must prioritize rigorous clinical trials to bridge this translational gap.

#### 4.3.3 Treating recurrent aphthous ulcers with hydrogels

**FIGURE 13:**
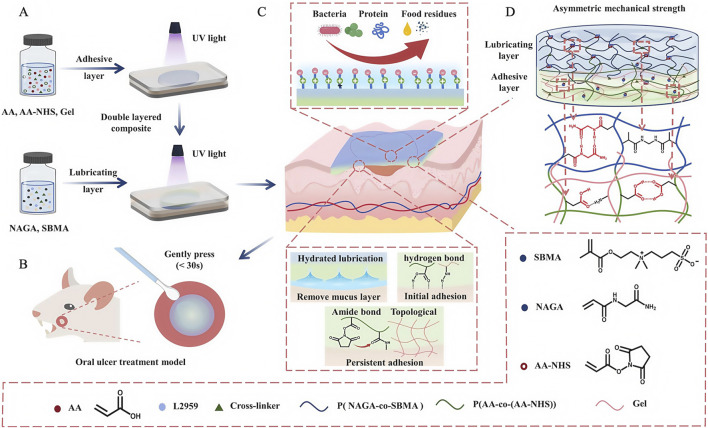
Development and medical use of the ANSB patch, utilizing lubrication and adhesion mechanisms derived from its asymmetric network structure. **(A)** Diagram showing the steps involved in creating the ANSB network. **(B)** ANSB patch diagram applied for oral ulcer treatment in rats. **(C)** Graphic illustrating the non-symmetrical mechanism of the zwitterionic hydrogel and the ionic cross-linking configuration. **(D)** The structure and cross-linking of the ionic network within the ANSB patch. **(A–D)** (Zhang et al., 2024) with permission from Elsevier.

Oral ulcers (OUs) are one of the most common diseases in the oral mucosa (Dudding et al., 2019); they cause severe pain and prevent patients from eating, chewing, and speaking, thereby affecting quality of life. Physiologically, due to a loss of mucosal barrier function and complex flora in the oral cavity, OU healing times are prolonged, while the disease shows periodic and recurrent characteristics. Therefore, recovering mucosa barrier functions using wound dressings is key for OUs. However, traditional dressings do not stably adhere in humid and hot environments in the oral cavity, while cheek and tongue mobility, masticatory jaw movements, and possible oral hygiene problems can further exacerbate dressings. Conversely, excellent water retention, wet adhesion, and biocompatible properties of hydrogels can compensate for traditional dressing disadvantages, thereby shortening treatment times and improving therapeutic effects for OUs. However, traditional hydrogel dressings have limitations: poor mechanical durability, long-term adhesion, and antifouling issues (Zhang et al., 2025). To further enhance hydrogel dressing properties, Zhang et al. (2024) exploited the multi-layered asymmetric construction of the natural mucosa and developed Janus patches (ANSBs). Patches integrated soft wet adhesive layers and resilient lubricating layers, which consisted of polymer chains formed by a gel and acrylic acid (Figure 13). The authors used N-hydroxysuccinimide ester (NHS) esters to catalyze adhesive layers, forming binding interfaced between these layers and tissues, linked by amide bonds and prolonging dressing retention times. Lubrication layers were formed by copolymerization with N-acryloylglycine (NAGA) and sulphonamide methacrylate (SBMA), which effectively reduced the lubrication protection of the natural mucosa, where the lubrication of SBMA also allows the dressing to exhibit both adhesive and anti-adhesive properties (Xing et al., 2022), addressing the issue that patches may adhere non-selectively to normal tissue, while NAGA with multiple hydrogen bonds also increases the strength of the lubrication layer on a simulated basis. In performance studies, ANSBs showed fast (≤30 s), powerful (≥45 kPa), and persistent (≥8 h) adhesion to moist oral tissues, whereas in animal studies, the control group retained slight redness and swelling in ulcer wounds and surrounding mucosal tissues on day 7; however, ulcer wounds in the ANSB-treated group recovered completely. Wound dressings like ANSB, which mimic the protective functions of the natural mucosal reconstruction barrier, have provided new therapeutic avenues for OU treatments.

In addition to new dressings developed using optimized physical and chemical hydrogel properties, hydrogel-based medicated dressings have also been used to treat OUs (Wang et al., 2024). Currently, topical drug therapy is the first-line OU treatment, mainly using topical corticosteroids (triamcinolone acetonide), anti-inflammatory drugs, and antibiotics (doxycycline) (Lau and Smith, 2022), but these commonly used drugs elicit side effects. For example, corticosteroids may affect immune and cardiovascular functions, while resistance develops with prolonged antibiotics use. Procatechol (PA) is a natural phenol extracted from herbs, has few side effects, and has antimicrobial and anti-inflammatory traits that accelerate wound healing (Wang et al., 2024a). In recent research, PA coordination with trivalent iron ions was used to form a tricomplex which was combined with quaternary ammonium deacetylated chitosan and aldehyde functionalized hyaluronic acid to form adherent hydrogel dressings (Wang et al., 2024b). PA in dressings promoted healing processes by inhibiting inflammation, promoting cellular proliferation, and inducing epithelial-mesenchymal transition, while aldehyde-functionalized hydrogel carriers exhibited adjustable mechanical features, self-healing capacity, and wet-adhesion, allowing for their retention in the oral cavity for longer periods, and releasing PA when compared to other hydrogel-based dressings. However, current research on PA and hydrogel combinations has only been applied to skin wounds (Liang et al., 2022). In the future, the therapeutic qualities of hydrogel dressings, such as the aforementioned combinations, should not be limited to OU treatments, but be expand to the therapeutic treatment of oral mucosal diseases.

From this evidence, we observed that different hydrogel dressings have corresponding mechanisms that promote healing in ulcer surfaces, such as rebuilding mucosal barriers, inhibiting infections, and limiting the extent of such infections. Hydrogel material properties, such as adhesion, water retention, drug delivery, and biocompatibility, can be exploited to provide new ideas for the clinical treatment of OU using hydrogel dressings.

## 5 Summary and prospects

As an excellent biomedical material with a unique 3D structure, hydrogels have been widely used to treat periodontal tissue and oral mucosal diseases. In this comprehensive review, we first systematically introduced the development status and clinical treatment methods for common diseases in periodontal soft, periodontal hard, and oral mucosal tissues. We then reviewed the application and development of hydrogels and systematically examined degradation rates and biocompatibility in clinical applications. Finally, we discussed the working principles and therapeutic effects of hydrogels in treating different periodontal soft tissue diseases, periodontal hard tissue diseases, and oral mucosal diseases.

Although hydrogels are used in many medical and clinical settings, we still face considerable challenges dealing with oral environmental conditions. For example, hydrogels carry drugs to oral environments and elicit treatment effects; however, many difficulties remain in balancing drug compatibility and the mechanical effects of hydrogels. Also, poor hydrogel degradation rates are also an issue. Another issue is the moist and dynamic oral cavity environment, which influences hydrogel adhesion. Furthermore, due to individual biological/immunological differences, patients may have different immune reactions to hydrogels; such reactions may not only affect treatment efficacy but may damage patients’ tissues. Finally, due to insufficient long-term biological safety profiles, low degradation rates, and high production costs, the clinical translation of hydrogel therapies for oral diseases has been slow, with many trials still at animal testing stages. Thus, accelerating the clinical translation of hydrogel therapy is our goal.

Despite drawbacks and challenges, many opportunities remain for future hydrogel development and applications. Breakthroughs in hydrogel research will depend on innovate and precise drug delivery and regulation, structural engineering technologies, and standardized evaluation systems. Through multidisciplinary collaborations, hydrogels can be transformed into safe, efficient, and ethically sound oral treatment therapies, translating from innovative development in the laboratory to clinical settings (Table 2).

**TABLE 2:** Comparison of hydrogel properties in dental soft and hard tissue diseases.

Disease	Hydrogel	Major polymers	Adhesion degree	Carrying ingredients	Drug-polymer interaction	Release mode	Reference
Periodontitis	LRG-P@*LGG*	PEGDA/LAP	Not mentioned	*LGG*	Physical mixing	Sustained release	Li K et al. (2024)
	PDMO hydrogel	PVA/DOPA	High (catechol modification)	Nanozyme + MnO_2_ NPs	Physical embedding	Light control	Hu et al. (2023)
	MN patch	GelMA	High (penetrating tissue)	Tetracycline-loaded poly (lactic-co-glycolic acid) NPs + cytokine-loaded SiMPs	UV crosslinking	Multistage sustained release	Zhang et al. (2022)
Periodontitis with DM	PBA-PE/OD hydrogel	OD/PBA-PEI	High (π - π interaction)	Doxy + MET	Covalent crosslinking	ROS response	Zhao X et al. (2022)
	GOE hydrogel	OHA/GelMA	Moderate (UV crosslinking)	self-assembled NPs (CHX/EGCG)	UV crosslinking	PH-response sustained release	Ge et al. (2024)
	Agevgel	AgNCs-Y/L monomer	Not mentioned	M2EVs	Covalent crosslinking	Sustained release	Peng et al. (2024)
	SZP/CS/β-GP hydrogel	CS/β-GP	Not mentioned	SZP	Physical embedding	Optical-Thermal response	Liu D et al. (2025)
Alveolar bone defect	DPSC-Exo/CS	CS/β-GP	Not mentioned	DPSC-Exos	Physical mixing	Sustained release	Shen et al. (2020)
	Gel-Alg Hydrogel	SA/Gel	Not mentioned	PDLSCs-Exos	Physical mixing	Not mentioned	Zhao Y et al. (2022)
	GH	CEC/HA-ALD/ADH	Not mentioned	nHA	Physical embedding	Sustained release	Pan Y et al. (2020)
OLP	DenTAl	Two layers: an adhesive surface a dissipative matrix	Extremely high (multicomponent adhesion)	Clobetasol-17-propionate	Physical entrapment	Sustained release	Wu D. T. et al. (2023)
	Poloxam spray hydrogel	Huangyuan gum Poloxam	Moderate (mucoadhesion)	HP-β-CD	Physical mixing	Thermoresponsive	Diaz-Salmeron et al. (2021)
OSF	BG/HA	Hyaluronidase	Not mentioned	Silicate ions	Physical entrapment	Sustained release	Guo et al. (2023)
	NanoCubogel	Lipid cubic phase hydrogel	High (SA modifying)	EGCG	Physical entrapment	Sustained release	Mehta, et al. (2021)
	HNC	Biodegradable PLGA NPs	High (SA modifying)	EGCG	Physical entrapment	Sustained release	Mehta, et al. (2024)
OU	ANSBs	AA/NAGA/SBMA	Extremely high (linked by amide bonds)	None	Not mentioned	Not mentioned	Xing et al. (2022)
	AHPs	QCH/aHA/PA-Fe^3+^	High (aldehyde-functional-ized)	PA	Ion coordination	Sustained release	Wang et al. (2024b)

Abbreviations: PEGDA, 4-arm PEG dimethacrylate; LAP, lithium phenyl-2, 4,6-trimethylbenzoylphosphinate; *LGG*, *Lactobacillus* rhamnosus GG; PVA, polyvinyl alcohol; DOPA, 3,4-dihydroxy-d-phenylalanine; NPs, nanoparticles; GelMA, gelatin methacryloyl; SiMPs, silica microparticles; OD, oxidised dextran; PBA-PE, phenylboronic acid-functionalised poly (ethyleneimine); Doxy, doxycycline; MET, metformin; OHA, oxidized hyaluronic acid; CHX:chlorhexidine acetate; EGCG, Epigallocatechin-3-gallate; AgNCs-Y, AgNCs–Y DNA, monomer; L monomer, cross-linker DNA monomer; M2EVs, M2 macrophage-derived extracellular vesicles; CS, chitosan; β-GP, β-sodium glycerophosphate; SZP, simvastatin@zeolitic imidazolate framework-8@polydopamine nanoparticles.

DPSC-Exos, dental pulp stem cell-derived exosomes; SA, sodium alginate; Gel, gelatin; PDLSCs-Exos, periodontal ligament stem cells-derived exosomes; CEC, N-carboxyethyl chitosan; HA-ALD, hyaluronic acid-aldehyde; ADH, adipic acid dihydrazide; nHA, nano-hydroxyapatite; HP-β-CD, hydroxypropyl-beta-cyclodextrin; PLGA, Poly (lactic-co-glycolic acid); AA, acrylic acid; NAGA, N-acryloylglycine; SBMA, sulphonamide methacrylate; QCH, quaternary ammonium deacetylated chitosan; aHA, aldehyde functionalized hyaluronic acid; PA-Fe^3+^, protocatechualdehyde and Fe^3+^; PA, procatechol.
